# A new bactericidal chlorinated derivative containing 2-aminooxazole potentiates antibacterial action of colistin against multidrug-resistant *acinetobacter baumannii*

**DOI:** 10.1007/s00430-025-00854-y

**Published:** 2025-09-19

**Authors:** Adéla Diepoltová, Daria Elzbieta Nawrot, Ondřej Janďourek, Martin Juhás, Pavel Bárta, Pavlína Vávrová, Vinod Sukanth Kumar Pallabothula, Paulína Dudášová-Hatoková, Marcela Vejsová, Barbora Voxová, Jan Österreicher, Petra Štěrbová-Kovaříková, Petr Nachtigal, Jan Zitko, Klára Konečná

**Affiliations:** 1 Faculty of Pharmacy in Hradec Králové, Charles University, Akademika Heyrovského 1203, 500 03 Hradec Králové, Czech Republic; 2 Department of Chemistry, Faculty of Science, University of Hradec Králové, Rokitanského 62, 500 03 Hradec Králové III, Czech Republic

**Keywords:** *Acinetobacter baumannii*, 2-aminooxazole, Anti-biofilm activity, Antimicrobial resistance, Checkerboard synergy study

## Abstract

This comprehensive study provides insight into the antibacterial action of a recently published 2-chloro-*N*-(oxazol-2-yl)isonicotinamide (AB15), intending to assess its potential as a candidate adjuvant molecule to support existing antibacterial drugs. Within the determination of the antibacterial effect, a promising activity against a member of the ESKAPE group with reduced treatment options, biofilm producer, *Acinetobacter baumannii*, was recognized (MIC of AB15 ranged from 15.63 to 62.5 µM). In addition, AB15 exhibited bactericidal activity and non/low-toxicity *in vitro* (IC_50_ > 1000 µM using HK-2 cells) and *in vivo* (LD_50_ > 500 mg/kg of body weight of the *Galleria mellonella* larvae, for both intra-hemocoel and per oral administration routes). Checkerboard assay revealed additive and synergistic interactions of AB15 and last-resort antibiotic drug, colistin (CST). Moreover, attention was also given to a frequently overlooked antibiofilm activity — the ability to suppress bacterial dissemination from microbial biofilms, and parameter MBDC (minimum biofilm dissemination concentration) was introduced. The study of the antibiofilm activity of AB15 and CST, both acting individually, or in AB15 + CST combination, revealed that AB15 has significant potential to suppress bacterial dissemination from biofilm formed by a clinical isolate *Acinetobacter baumannii* and that it contributes to this effect when combined with CST. Finally, AB15 + CST combination demonstrated significantly greater biocompatibility towards human erythrocytes than CST acting individually at an equivalent antibiofilm-effective concentration. The role of AB15 as a promising adjuvant molecule to CST is also supported by its distinct mechanism of action, which reduces the risk of antimicrobial resistance emergence. To conclude, AB15 exhibits several essential attributes that support its designation as a promising antibiotic adjuvant.

**Graphical abstract:**

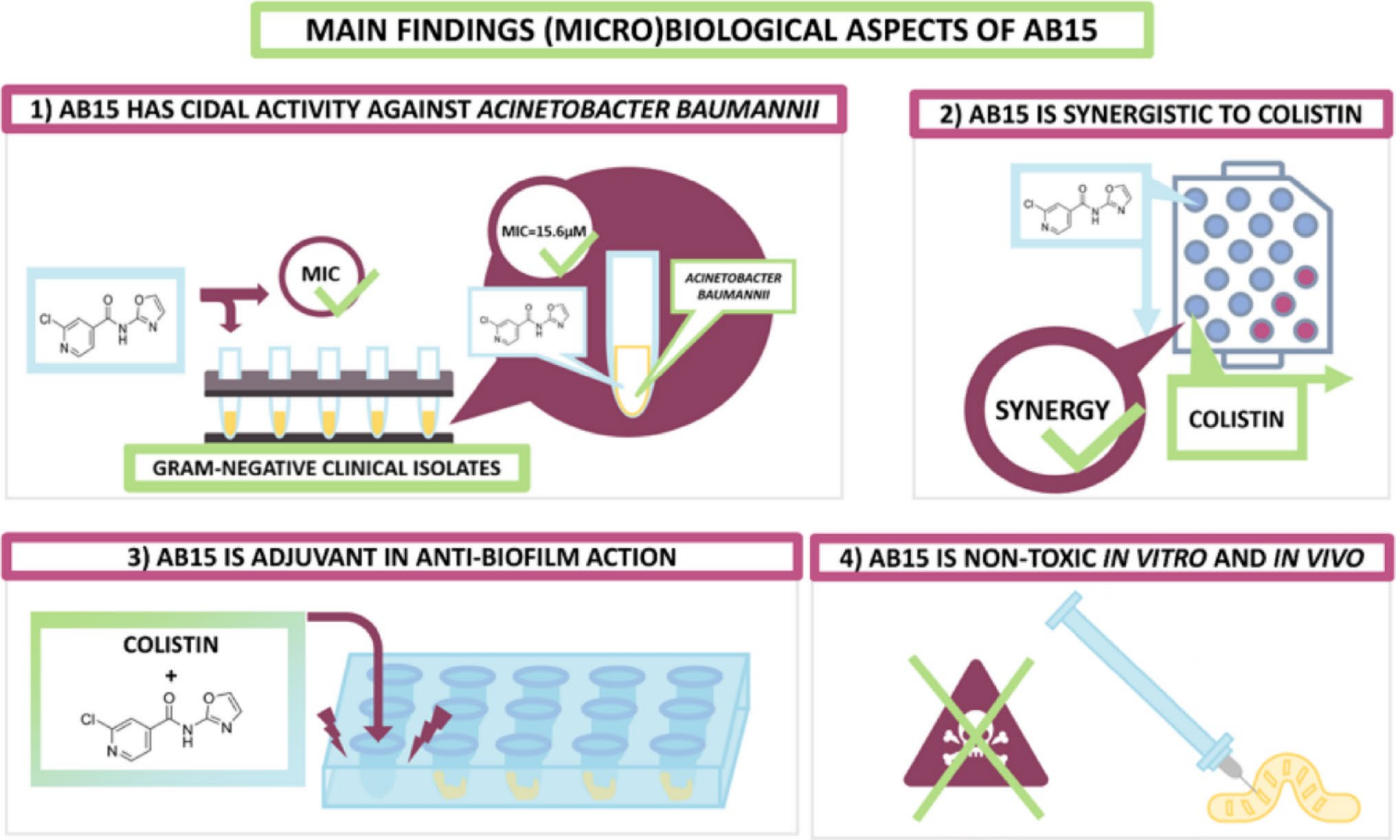

**Supplementary Information:**

The online version contains supplementary material available at 10.1007/s00430-025-00854-y.

## Introduction

Human society is suffering from a long-lasting crisis of antimicrobial resistance (AMR) that is quickly escalating due to an extreme lack of effective antimicrobial substances [1, 2]. Misuse and overuse of antibiotics, excessive application of anti-infectives in agriculture, insufficient compliance of patients with antibiotic guidelines and poor hospital surveillance have all undoubtedly contributed to this situation [3]. Global society is being challenged by the consequences of the high rate of AMR, which leaves a scar in a form of serious socio-economic issues [4, 5].

The AMR crisis has been threatening the population for years, and the problem does not seem to be slowing down. In 2017, the World Health Organization (WHO) published a list of bacterial pathogens with a high-risk status, against which an urgent increase in antimicrobial drug research is desperately needed [6]. In 2024, the list of priority bacterial pathogens has undergone some changes. However, the ongoing urgent call for developing new antimicrobials is still reflected in the current WHO Global Research Agenda for AMR [7, 8].

Microorganisms themselves are non-negligible contributors to this stalemate situation. They possess a wide range of different resistance mechanisms and strategies to help them exist in hostile environments [9]. With regard to the medical importance and alarming stage of resistance, extraordinary attention has been recently given to the group of bacteria contained under the ESKAPE acronym (*Enterococcus faecium*, *Staphylococcus aureus*, *Klebsiella pneumoniae*, *Acinetobacter baumannii*, *Pseudomonas aeruginosa*, and *Enterobacteriaceae* family*)*. ESKAPE bacteria show a high rate of AMR against the majority of common antibiotic groups including penicillins, cephalosporins, macrolides, oxazolidinones, lipopeptides, fluoroquinolones, tetracyclines, and even against antibiotic groups such as carbapenems, glycopeptides, and polymyxins, which are listed as the last line of defense. In addition, these multidrug-resistant (MDR) opportunistic pathogens have been recognized as biofilm-producing bacteria, capable of colonizing biotic tissues and abiotic medical devices such as catheters, prosthetic joint replacements, and endotracheal tubes. This ability contributes to the fact that ESKAPE bacteria represent important causative agents of nosocomial infections that have extremely reduced treatment options and show increased mortality [10–12].

Generally, the formation of biofilm microbial consortia is recognized as a virulence factor underlying especially chronic infections. The ability to form biofilm multicellular consortia expands the portfolio of resistance mechanisms and offers new protection options for participating cells [13–17].

One of the key factors contributing to the significantly higher resistance (up to 1000 times) of biofilm-forming bacteria compared to their planktonic form lies in the complex heterogeneous architecture of the whole biofilm consortium and the presence of extracellular matrix (ECM) [18]. ECM comprises substances produced by participating cells, such as polysaccharides, proteins, lipids, and nucleic acids. ECM represents a mechanical barrier that limits the permeability for some antimicrobial drugs. However, the antimicrobial activity of drugs capable of diffusion through ECM can be limited or inactivated by the structural components of ECM, especially by microbial enzymes [19]. In addition, the lower stages of the three-dimensional architecture of the biofilm consortia are inhabited by cells with a dormant phenotype. Due to their low-to-none metabolic activity, these cells become invulnerable to the effect of antimicrobial drugs that affect target microbial structure(s) operating only in metabolically active cells [20, 21].

Gram-negative bacterium *Acinetobacter baumannii* (*A. baumannii*), an infamous member of the ESKAPE group, reigns at the top of the worrisome list of resistant pathogens with the highest priority. *A. baumannii* brings a real challenge to clinicians as a leading cause of life-threatening nosocomial infections such as pneumonia and bloodstream infections, especially in immunocompromised patients hospitalized in intensive care units [22]. The severity of the situation is demonstrated in a study published by Chen *et al.,* 2017, where it is stated that 73.6% out of 403 different clinical isolates of *A. baumannii* revealed resistance to quinolones, 71.3% to sulfonamides and more than 50% to cephalosporins, combination of beta-lactam/beta-lactamase inhibitors, and carbapenems [23]. As indicated in a study conducted by Nishimura *et al*., 2022, it was shown that carbapenem-resistant *A. baumannii* strains isolated from patients with pneumonia corresponded to 84 out of 86 (97.67%) [24].

Several strategies appear to be focused on this effort. Searching for a new solitary antibiotic molecule is usually time-consuming and economically challenging. Therefore, within the effort to accelerate the process, non-toxic structures with less significant activity but additive, beneficial effects to the commercially established drugs cannot remain overlooked. These added values are met by the inclusion of antibiotic adjuvants. Adjuvants mostly represent molecules with low or no antimicrobial activity that synergize, restore, and conserve the activity of antibiotics and prevent the spread of resistance [25].

In this comprehensive study, we focused our attention on 2-chloro-*N*-(oxazol-2-yl)isonicotinamide, designated AB15. In our previous study [26], we revealed some favorable physico-chemical and biological properties based on which we decided to subject AB15 to a more extensive and detailed study focused especially on essential attributes for antibacterial drug candidates or valid candidate adjuvant molecules.

## Materials and methods

### Chemistry

The compound was synthesized and characterized as published previously [26]. The identity and purity were checked using nuclear magnetic resonance spectroscopy (^1^H NMR and ^13^C NMR), infrared spectroscopy (IR), high-resolution mass spectroscopy (HRMS), and high-performance liquid chromatography (HPLC). The purity was > 95%. The analytical data, including the NMR spectra (Fig. S1) and HPLC chromatograms (Fig. S2), instrumentation, and methodology are located in Supplementary Information.

### Determination of *in vitro* antibacterial activity of AB15 against clinical isolate bacterial strains

The bacterial clinical isolates were provided by the Department of Clinical Microbiology, University Hospital, Hradec Králové, Czech Republic. Each strain was taxonomically classified by biochemical tests and MALDI-TOF (Microflex LH/SH MALDITOF, Bruker Biotyper 3.0 SW, Bruker Daltonic GmbH, Bremen, Germany) instrumentation. The antibiograms of the strains were determined using the disc diffusion methodology according to The European Committee on Antimicrobial Susceptibility Testing (EUCAST) recommendations [27] or the microdilution broth method according to EUCAST with slight modifications [28]. The cultivation was performed in Cation-adjusted Mueller–Hinton broth (CAMHB, M-H 2 Broth, Merck, United States) at 35 ± 2 °C. The candidate compound AB15 was dissolved in DMSO (Merck, United States) to produce stock solution. The final concentration of DMSO in the testing medium did not exceed 1% (v/v) of the total solution composition and did not affect the growth of bacteria. Both positive growth (microbe alone in cultivation medium and 1% DMSO, v/v) and negative growth (cultivation medium and 1% DMSO, v/v) controls were involved in all assays. Internal quality standards ciprofloxacin (CIP, Merck, United States) and gentamicin (GEN, PanReac AppliChem, United States) were employed in assays as well (Table S1, Supplementary Information). Antibacterial activity expressed as minimum inhibitory concentration (MIC, reported in µM or mg/L) was evaluated after 24 h of static incubation in a dark and humidified atmosphere at 35 ± 2 °C. Visual inspection and spectrophotometric measurements were engaged to evaluate the MIC endpoints (530 nm, Synergy HTX Multi-Mode Microplate reader, BioTek, United States).

### Distinguishing between bactericidal and static effect of AB15

To distinguish between the bactericidal or bacteriostatic activity of AB15, the clinical isolate strain, *A. baumannii* (20/21), was employed in microdilution broth method, according to a methodology mentioned above and consequent spread plate method was used to calculate colony forming units (CFU). The antibacterial agent is usually regarded as bactericidal if the MBC is not higher than four times the MIC. After the incubation for 24 h at 35 ± 2 °C, the MIC was read, and the representative aliquots were taken from wells with registered growth inhibition corresponding to MIC–4 × MIC. Aliquots were subsequently serially diluted and subcultured on Mueller–Hinton agar for 24 h in a humid atmosphere at 35 ± 2 °C. Similarly, the initial bacterial inoculum was processed. After the incubation period, the number of CFU/mL was calculated, and it was assessed whether the criterion corresponding to the MBC had been met. MBC is defined as the lowest concentration of antimicrobial agent that leads to the reduction of initial bacterial inoculum viability corresponding to a value ≥ 99.9%.

### Determination of the mechanism of action of AB15 using macromolecular biosynthesis assay

Briefly, methicillin-resistant *Staphylococcus aureus* subsp. *aureus* (MRSA, American Type Culture Collection, ATCC 43300) grown on Tryptic Soy agar (Merck, USA) was transferred into Tryptic Soy Broth (TSB, Merck, USA) and cultured overnight. A completely defined medium (CDM) / CDM-Leu (for determination of protein synthesis pathway) prepared according to the study published by Nowakowska, *et al*., 2013 [29] was used for the preparation of log-phase culture of MRSA (approx. 2 × 10^7^ CFU). TSB culture was diluted with these media in ratio 1:100 and cultured at 37°C for another 5 h. 0.9 mL of suspensions was transferred into pre-warmed glass tubes. All antimicrobials and the tested compound (vancomycin (VAN) = 8 mg/L, rifampicin (RIF) = 0.064 mg/L, CIP = 1 mg/L, chloramphenicol (CHL) = 64 mg/L, chlorhexidine (CHX) = 4 mg/L, and AB15 = 56 mg/L) were added at a final concentration equal to 4 × MIC and mixed thoroughly. All standards were purchased from Merck (Merck, USA). Untreated controls, bacteria in CDM medium with 1% (v/v) DMSO were prepared along with other samples. [^3^H]-labelled precursors (*N*-acetylglucosamine–0.1 µCi/mL, uridine–1 µCi/mL, thymidine–1 µCi/mL, leucine–3 µCi/mL) (Hartmann Analytic, Germany) were immediately added to corresponding tubes. Another incubation at 37 °C for 2 h followed. 0.5 mL aliquots were transferred into 2 mL centrifuge tubes containing 1 mL of ice-cold 10% trichloroacetic acid (TCA, Merck, USA), mixed thoroughly, and placed on ice overnight to facilitate the precipitation. The precipitates were then washed once with 0.5 mL of 5% TCA/1.5M NaCl followed by one-time washing with 0.5 mL of 5% TCA to remove free precursors. After the second wash, samples were solubilized in 0.5 mL of 0.1% SDS/0.1M NaOH by vortexing at room temperature. The solubilized precipitates were transferred into scintillation tubes and thoroughly mixed with 2 mL of scintillation cocktail (Merck, USA). The radioactivity of incorporated precursors was measured in counts per minute using a liquid scintillation analyzer TRI-CARB 2900TR (Perkin Elmer, USA). The results were expressed as a percentage related to untreated controls (100%).

Data were analyzed using GraphPad Prism 9.0.0 (GraphPad Software, Inc., USA). One-way analysis of variance (ANOVA) was used to determine the statistical significance (*p*) of differences in this* in vitro* assay.

### Determination of the impact of AB15 on bacterial cytoplasmatic membrane

A voltage-sensitive dye, DiSC3(5), was employed in a fluorometric measurement of membrane potential to detect the possible depolarizing effect of AB15 on bacterial cytoplasmic membrane. As the reference bacterial strain, MRSA (ATCC 43300), was used in this assay.

The MRSA strain was resuspended in CAMHB medium and cultivated until the exponential (mid-log) phase was reached. After centrifugation (10,000×*g*, for 10 min at 24 °C) and washing steps, the bacteria were resuspended in 5 µM 4-(2-hydroxyethyl)-1-piperazine ethanesulfonic acid (HEPES) with 5 µM glucose (pH 7.2) and diluted to optical density (O.D.) = 0.5 McFarland units. The voltage-sensitive dye DiSC3(5) was then added in a final concentration of 0.5 µM (1% DMSO, v/v) and the suspension was incubated for 15 min. After that, the suspension was transferred to a white polystyrene 96-well plate in the volume of 200 µL/per well and a fluorescence quenching (λ_Ex_ = 620 nm, λ_Em_ = 680 nm, Synergy HTX Multi-Mode Microplate reader, BioTek, USA) was monitored for 10 min until a stable baseline was obtained. Membrane-active chlorhexidine (CHX, positive control) was employed as the positive control, in a final concentration of 4 mg/L and 1% DMSO (v/v). The tested compound, AB15, was added in a final concentration corresponding to 4 × MIC (4 × 28) mg/L, with 1% DMSO (v/v). Samples were added to the wells with bacterial suspension in hexaplicates at a final concentration of 1% DMSO (v/v)*.* The negative control included untreated bacterial cells in the HEPES buffer (1% DMSO, v/v). Fluorescence was continuously monitored every 1 min for the next 35 min.

### Evaluation of *in vitro* cytotoxicity using HK-2 cells

The human kidney epithelial cell line (HK-2, ATCC, USA) was cultured in Dulbecco´s Modified Eagle's Medium–high glucose (Sigma-Aldrich, USA) supplemented with 10% fetal bovine serum (Sigma-Aldrich, USA) and 1% L-glutamine solution (Sigma-Aldrich, USA) in a humidified atmosphere containing 5% CO_2_ at 37 °C.

The HK-2 cells were seeded in a density of 10 000 cells per well in a 96-well plate 24 h before the experiment. The next day, the cells in triplicates were treated with the tested substance at a range of concentrations (1–1000 µM). The controls representing 100% cell viability, 0% cell viability (the cells treated with 10% DMSO), no cell control, and vehiculum controls were incubated in parallel. The plate was incubated for 24 h in a humidified atmosphere containing 5% CO_2_ at 37%. After the incubation, the reagent from the kit CellTiter 96 AQueous One Solution Cell Proliferation Assay (CellTiter 96; PROMEGA, Fitchburg, USA) was added. After 2 h incubation at 37 °C, the absorbance of samples was recorded at 490 nm (TECAN, Infinita M200, Austria). A standard toxicological parameter, half maximal inhibitory concentration (IC_**50**_) was calculated by nonlinear regression from a semi-logarithmic plot of incubation concentration versus percentage of absorbance relative to untreated controls using GraphPad Prism 9.0.0 (GraphPad Software, Inc., USA).

The results of the experiments are presented as inhibitory concentration, which reduces the viability of the cell population to 50% from the maximal viability (IC_**50**_). The IC_**50**_ values were calculated in GraphPad Prism 9.0.0 (GraphPad Software, Inc., USA) for the tested compound in the concentration range 1–1000 µM.

### Screening of *in vivo* toxicity using the invertebrate animal model Galleria mellonella

*Galleria mellonella* larvae were reared in the laboratory of the Department of Biological and Medical Sciences (Microbiology and Immunology Section), at the Faculty of Pharmacy in Hradec Králové, Charles University. The model animals were fed with an artificial diet according to Haydak *et al*., 1936 [30] and kept in the dark at 29 °C. Only fully vital, cream-colored larvae with the weight ranging from 280 to 320 mg were selected for tested compound administration for each experiment. AB15 was dissolved in DMSO and diluted with a phosphate buffer saline, pH 7.4 (Merck, USA) to the required working concentration. The final concentration of DMSO corresponded to 30% (v/v). The samples were administered into the hemocoel through the last left proleg using a Hamilton syringe at a total volume of 10 µL/per larva. For the per oral administration route, the force-feeding method was employed. In each experiment, two control groups (untreated control and control with administered 30% DMSO (v/v) in PBS) were included. The inoculated larvae and control groups were then incubated in Petri dishes at 37 °C. The survival and health conditions of larvae were recorded over a 120 h period (24, 48, 72, 96 and 120 h after administration). Death was defined as the complete loss of mobility, including a physical stimulus using a plastic pipette.

The mortality for each dose/time interval was calculated, and survival Kaplan–Meier curves were designed via SW analysis (GraphPad Prism 9.0.0, GraphPad Software, Inc., USA). Screening survival experiments within *in vivo* toxicity studies consisted of 78 individuals. For evaluation of toxicity after intra-hemocoel administration, 48 individuals were included (*n* = 8). In the assessment of toxicity after per oral administration, 30 individuals were included (*n* = 5). Data from the survival experiments were subjected to the log-rank Mantel-Cox (curve comparison) test and the Mantel–Haenszel (hazard ratios) pairwise comparison test. The results were considered significant at a *p-*value < 0.05 in all analyses.

### Checkerboard assays

Synergy measurements were procured with the use of the checkerboard assays. This microdilution-based method was employed as a screening of a mutual antibacterial effect within the interaction of two compounds with antimicrobial activity (AB15 vs. selected antibacterial drugs). The assays were performed in Honeycomb plates (Oy Growth Curves, Finland) in a ten-by-ten well configuration. CIP (Merck, United States), GEN (PanReac, AppliChem, United States), tigecycline (TGC, Acros Organics, Belgium), trimethoprim-sulfamethoxazole (SXT, Merck, United States), colistin (CST, Cayman Chemical, United States) and CHL (MP Biomedicals, United States) were selected for the combinations. The *Escherichia coli* (*E. coli*) ATCC 25922 reference strain was used for detecting the total antibacterial activity of the compounds in combinations. To produce stock solutions, the compounds were dissolved in DMSO (Merck, United States) and then serially diluted in CAMHB in separate microtiter wells and then transferred to a Honeycomb plate at a 1:1 volume ratio. AB15 was transferred in vertical and selected antibacterial drug in horizontal direction. The ranges of concentration ratios were selected and optimized based on previous evaluations of the MIC for the individual compounds. The final concentration of DMSO in the testing medium did not exceed 1% (v/v) of the total solution composition and did not affect the growth of bacteria. Each well with a final volume of 200 µL of compound mixtures was inoculated with 10 µL *E. coli* reference strain. The positive growth controls consisted solely of the testing microbe in CAMHB with 1% (v/v) DMSO, and the negative growth controls consisted of CAMHB and 1% (v/v) DMSO only. The honeycomb plates were incubated in Bioscreen C instrument (Oy Growth Curves, Finland) at 36.8 °C for 20 h. At every 15 min of incubation, O.D. was recorded at 580 nm. After the incubation, the percentage of growth inhibition was calculated and compared to the positive growth controls (background values subtracted). A visual inspection and metabolic activity indicator, Alamar Blue (Alamar Blue TM Cell Viability reagent, Thermo Fisher Scientific, United States), were also employed for evaluating MIC endpoints. The total Fractional Inhibitory Concentration Index (FICI) was used to interpret the checkerboard assays results. The FICIs were calculated according to the following formula: FICI = FIC_A_ + FIC_B_. FIC_A_ and FIC_B_ were calculated as follows: Fractional Inhibitory concentration (FIC_A_ or _B_) = MIC of drug A (or B) in combination/MIC of drug A (or B) alone. The results of the FICIs were defined as follows: FICI ≤ 0.5—synergy; 0.5 < FICI ≤ 1—additivity; 1 < FICI ≤ 4—indifference; and FICI > 4—antagonism.

### *In vitro* screening of antibiofilm activity—recognition of a potential to inhibit bacterial dissemination of Acinetobacter baumannii from biofilm communities, and efficacy in targeting biofilm-embedded cells

The microtiter plate biofilm assay was employed to assess the parameters indicating the anti-biofilm activity of AB15, CST, and a combination of both compounds against a biofilm formed by clinical isolate of *A. baumannii.* 10^8^ CFU/mL culture of *A. baumannii* (20/21) was prepared in CAMHB containing 1% (w/v) glucose. 200 µL of the bacterial culture was then pipetted into each well of flat-bottom, polystyrene, non-tissue-treated microtiter 96-well plates. The negative growth control (only medium without any bacterial agents and DMSO) was included as well. After 24 h of static incubation in a dark and humidified atmosphere at 35 ± 2°C, the supernatant was decanted. Formed biofilms were washed three times with sterile 0.9% saline solution and let to air dry for 15 min at laboratory temperature. The stock solutions of the tested compounds were prepared in DMSO. Subsequently, solutions of AB15, and CST were prepared in CAMHB and with a final concentration 1% DMSO (v/v). Solutions with the final concentration of AB15 ranging from 3.9 to 2000 µM, and CST ranging from 0.054 to 27.694 µM in volume 200 µL/per well were added to preformed biofilms. For determination of the anti-biofilm activity of AB15 and CST in combination, the multiples of concentrations ranging from 1 × to 128 × MIC corresponding to the concentration ratio, 7.813 µM (AB15) + 0.108 µM (CST) were added to preformed biofilms. The concentration ratio was selected following previous results from the checkerboard study, where it was recognized as promising. All samples were therefore prepared in octaplicates. Positive growth control containing solely unexposed biofilms with 200 µL of CAMHB and negative growth control (CAMHB and 1% (v/v) DMSO only) were involved in the assay, as well. Afterward, the cultivation step for 24 h in a dark and humidified atmosphere at 35 ± 2 °C was included.

After the incubation, 190 µL of the supernatant was transferred to clean plates and a metabolic activity indicator, Alamar Blue, was added into each well. After 30 min of incubation (slow shaking mode included) at 35 ± 2 ◦C, a visual inspection together with fluorescence measurement (λ_Ex_ = 528 nm and λ_Em_ = 585 nm) using a plate reader (Synergy HTX Multi-mode reader, BioTek, USA) were performed.

There is an inconsistent perception of biofilm susceptibility parameters and definition for minimum biofilm inhibitory concentration in research studies [31]. It has been established that bacteria released from the biofilm consortia, transitioning from a biofilm-embedded state to a transient planktonic lifestyle, undergo phenotypic adaptation characterized by a shift to a metabolically active stage, thereby facilitating successful dissemination of infection and colonization of new environments [32, 33]. In our study, an overlooked antibiofilm action involving the suppression of metabolic activity of planktonic bacterial cells disseminated from biofilm communities was evaluated. Hence, the abbreviation MBDC (minimum biofilm dissemination concentration) is introduced in this study. The MBDC is defined as the minimum antimicrobial concentration resulting in 95% inhibition of the metabolic activity of biofilm-dispersed cells present in the medium after drug exposure to preformed biofilms relative to the positive growth control (drug unexposed biofilms). The release of these cells *in vivo *from biofilms represents the potential for spreading the biofilm-forming agent from infectious deposits. Thus, the inhibition of metabolic activity (associated with viability) of biofilm dispersed cells extrapolates the limitation of biofilm recolonization capacity in the host. Both, visual inspection and fluorescence measurement, were employed for the MBDC evaluation.

For the determination of Minimum Biofilm Eradication Concentration (MBEC), preformed *A. baumannii* biofilms were exposed to tested compounds in the final concentrations described above. After the cultivation step, biofilms were rinsed four times with sterile 0.9% saline solution and 200 µL of CAMHB medium was added to each biofilm-containing well. After that, sonification (5 min) and vortexing steps were included for biofilm disaggregation and restoration of metabolic activity of persistent cells. Finally, an alternative approach for evaluation the activity of tested compounds against biofilm-forming agents, using the Alamar Blue metabolic indicator was employed. 10 µl of the Alamar Blue were transferred into each well, and the plates were incubated for 30 min in slow shaking regime at 35 ± 2 ◦C, Afterward, a visual inspection together with fluorescence measurement (λ_Ex_ = 528 nm and λ_Em_ = 585 nm) using a plate reader (Synergy HTX Multi-mode reader, BioTek, USA) was performed. The MBEC corresponded to the lowest concentration of tested compound(s), resulting in 95% inhibition of metabolic activity of disaggregated biofilm-forming agents, relative to the positive growth control (drug-unexposed biofilms). Data were then compared using ANOVA in GraphPad Prism 9.0.0 (GraphPad Software, Inc., USA).

### Determination of hemolytic activity using ex vivo human red blood cell hemolysis assay

Blood samples were acquired from three human voluntary donors and subsequently pooled. Blood was then centrifuged (1000×g, 10 min), supernatants were discarded, and the pellets were washed three times with Hartmann’s medium. The final cell pellet was diluted in ratio 1:7 (v/v) with Hartmann’s medium. Further, solutions of CST alone and a combination of CST with AB15 were prepared in Hartmann’s medium. 0.5 mL of cell suspension was transferred into each test tube with 0.5 mL of drug(s) solutions. The final concentration of CST and the combination of CST + AB15 in hemolysis assay corresponded to the highest concentration established in antibiofilm activity assessment (previous chapter). For CST, the final concentration in hemolysis assay corresponded to 256 × multiplicity of MIC_CST_ (55.3 mM), and the final concentration of CST + AB15 combination corresponded to 16 × multiplicity of MIC_CST+AB15_ (1.728 µM CST + 125 µM AB15).

The mixture was kept at 37 °C for 1 h. After incubation, the cell suspension was centrifuged, and the supernatant was carefully collected. The amount of hemoglobin, released from red blood cells (RBC) into the supernatant, was monitored by measuring the absorbance at 405 nm [34, 35] with a spectrophotometer (Synergy HTX Multi-mode reader, BioTek, USA). The negative control (RBC in Hartmann’s medium only) and positive controls (RBC in Hartmann’s medium, sonicated by ultrasonic needle (Ultrasonic Processor UP100H, Hielscher Ultrasonic, Germany) for 1 min), were included as well. Data gathered in the determination of hemolytic activity experiments were subjected to ANOVA test in GraphPad Prism 9.0.0 (GraphPad Software, Inc., USA).

## Results and discussion

### The chlorinated derivative AB15 containing 2-aminooxazole shows promising attributes for a drug candidate

**Fig. 1:**
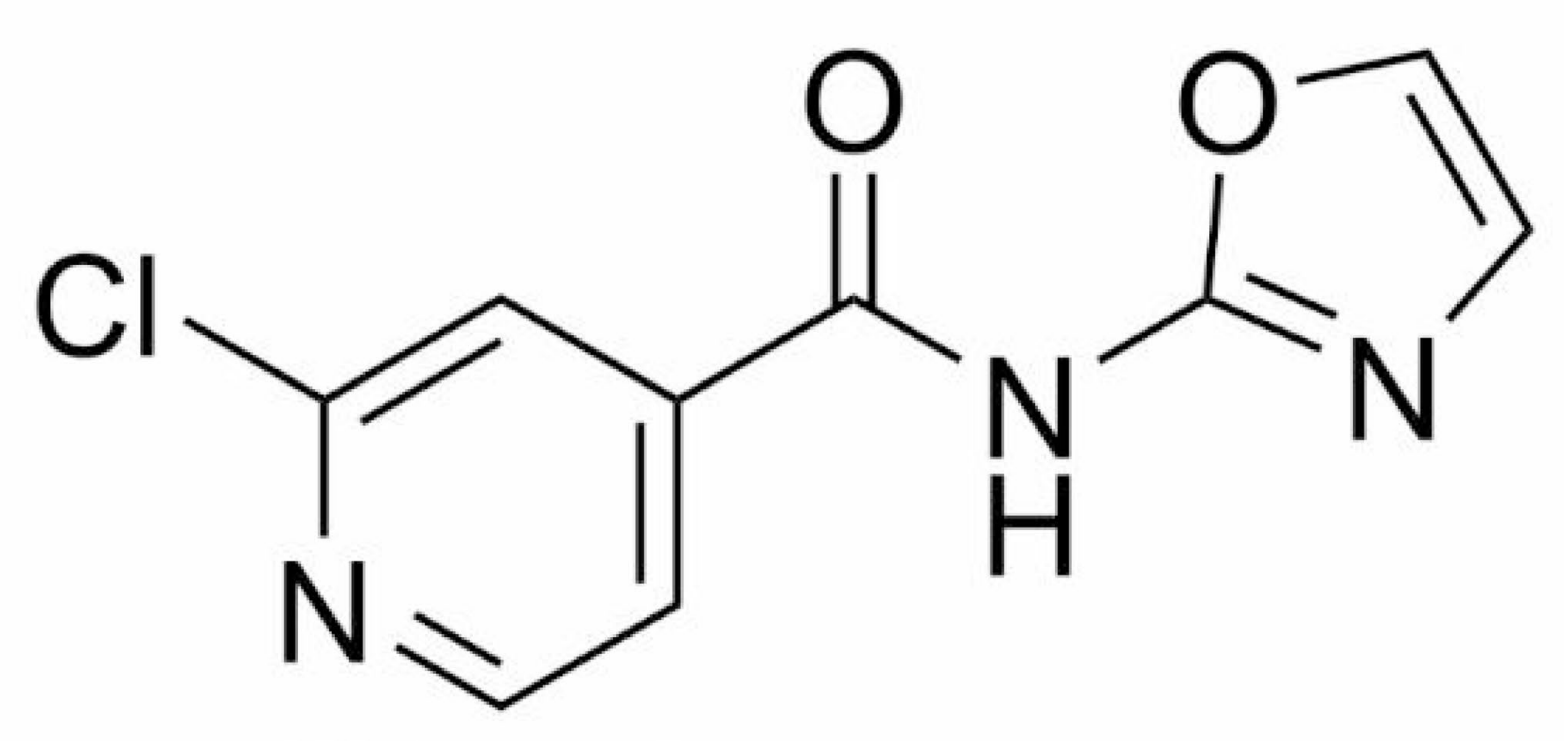
The chemical structure of the studied compound AB15

In our previous study published by Juhás *et al*., 2022 [26], we investigated the physico-chemical and some biological properties in a series of substituted *N*-oxazol-2-yl and *N*-thiazol-2-yl carboxamides. It has generally been concluded that oxazole-containing compounds have more favorable physico-chemical and biological properties. Based on our findings associated with some essential attributes for drug candidates such as solubility, *in vitro* cytotoxicity, sufficient stability, and promising antibacterial activity, we decided to subject the compound AB15 (2-chloro-*N*-(oxazol-2-yl)isonicotinamide; in the previous study designated as 6b, Fig. 1) to further investigation. This advanced study was intended to recognize whether this compound can be legitimately selected as a potential candidate adjuvant molecule for antibiotic therapy. Within the evaluation of a favorable profile for drug-likeness criteria by in silico approach, it can be stated that all the commonly applied physico-chemical rules were met e.g., the Lipinski, Veber, and Muegge rules (Table S2, Supplementary Information). AB15 also fulfills stricter lead-likeness criteria (Oprea), allowing the further structural modifications (Table S2). In silico prediction of pharmacokinetic parameters by SwissADME [36] (Table S2) indicates high gastrointestinal absorption and blood–brain barrier penetration based on the boiled-egg method [37]. AB15 is a small molecule with balanced numbers of polar and non-polar functional groups. Such fragments are valuable in medicinal chemistry instead of bulky and greasy molecules.Many successful antibacterial compounds tend to be more polar and less lipophilic, facilitating penetration through bacterial cell envelopes and avoiding efflux. This trend is especially important for compounds targeting Gram-negative bacteria [38].

AB15 is expected to be metabolically stable since the carboxamidic linker is sterically protected. This is confirmed by the metabolism prediction by BioTransformer 3.0 [39] (Phase I CYP450, combined model), which suggests mild oxidation reactions, mainly the *N*-oxidation of the pyridine nitrogen or hydroxylation of the heteroaromatic rings, but not the hydrolysis of the amidic linker (Table S3).

**Fig. 2:**
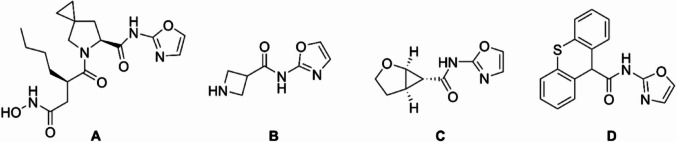
Examples of compounds bearing *N*-(oxazol-2-yl) moiety described in the literature

In an attempt to find similar compounds and their application, we performed a search of the existing literature. Surprisingly, no biological activity was reported for *N*-oxazol-2-yl)pyridinecarboxamides, or compounds with analogous simple (hetero)aromatic rings in the acyl part (benzene, thiophene, furane etc.). The acyl part of similar compounds with reported biological activity was either alicyclic (Fig. 2, compounds A, B, C) or larger heteroaromatic (Fig. 2D).

Compound A was investigated for antibacterial and antitumor activity [40], compound B was investigated as an antitumor agent [41], compound C was investigated for eliminating invertebrate pests [42], and compound D was investigated as a metabotropic glutamate receptor ligand [43].

**Fig. 3:**
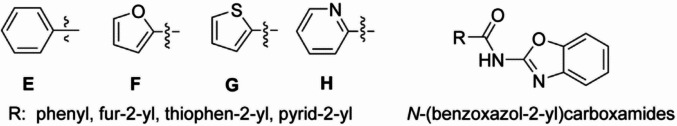
Examples of *N*-(benzoxazol-2-yl)carboxamides described in the literature

Several benzoxazole analogues, that is *N*-(benzoxazol-2-yl)carboxamides (Fig. 3), were investigated for antimicrobial activity. *N*-(Benzoxazol-2-yl)benzamide [44], *N*-(benzoxazol-2-yl)furan-2-carboxamide [45, 46], and *N*-(benzoxazol-2-yl)thiophene-2-carboxamide [45] were investigated as antimycobacterial compounds, often with activity against MDR and extensively drug-resistant (XDR) *Mycobacterium (M.) tuberculosis* strains.

Interestingly, *N-(*benzoxazol-2-yl)pyridine-2-carboxamide, similar in its acyl part to our compound, was tested as an apolipoprotein A-I expression stimulator [47]. Based on our literature search, we concluded that neither our compound nor a structurally similar one has been studied for antibiofilm properties.

### The promising activity of AB15 against some medically relevant bacteria from the ESKAPE group

As shown in our previous study [26] within the antibacterial activity screening of AB15 against four selected Gram-positive and four Gram-negative reference bacterial strains, the highest activity (after 24 h incubation) corresponding to 62.5 µM was revealed against MRSA (ATCC 43300), *Staphylococcus epidermidis* (ATCC 12228), *E. coli* (ATCC 25922) and *A. baumannii* (ATCC 19606) (Table S4, Supplementary Information). A significantly more pronounced antimycobacterial activity of AB15 corresponding to a concentration range of 13.95–27.90 µM was registered against a broad spectrum of mycobacteria (*Mycolicibacterium smegmatis, Mycolicibacterium aurum, M. avium, M. kansasii, M. tuberculosis* H37Ra, *M. tuberculosis* H37Rv and multidrug-resistant *M. tuberculosis* IZAK, *M. tuberculosis* MATI (Table S5, Supplementary Information). The highest antifungal activity of AB15 was registered against the yeast strain *Candida albican*s (ATCC 2443), corresponding to 31.25 µM, and filamentous fungus *Lichtheimia corymbifera* (Czech Collection of Microorganisms–CCM 8077), corresponding to 62.5 µM (Table S6, Supplementary Information).

### A follow-up study of the antibacterial activity of AB15—the introduction of Gram-positive and Gram-negative clinical bacterial isolates

Based on the results mentioned above, a set of highly medically relevant clinical bacterial isolates with determined antibiogram profiles was employed to provide a deeper insight into antibacterial action of AB15. Specifically, four *Staphylococcus* and twelve Gram-negative bacterial isolates were selected. As shown in Table S7 (Supplementary Information), the activity of AB15 against four *Staphylococcus* strains, including vancomycin-resistant ones, showed MICs ranging from 62.5 to > 250 µM. Members of the ESKAPE group with high resistance and MDR profiles were placed in an extended study of AB15 antibacterial action against Gram-negative bacteria. The susceptibility/resistance profiles of these strains, listed in Table S8 (Supplementary Information), were determined by disc diffusion and microdilution broth methods employed according to the recommendations of EUCAST. As it is shown in Table 1, the MIC ranged from 15.63 to > 500 µM. The highest activity (15.63 µM) was recognized against MDR *A. baumannii* (20/21), and the lowest against MDR *Pseudomonas aeruginosa* (26/21) (> 500 µM). The registered moderate activity of AB15 against *A. baumannii,* the bacterial agent mentioned as being among the most threatening in the list of antibiotic-resistant “priority pathogens” supported the strong premise of continuing in a more comprehensive characterization of other antibacterial activity attributes and for making easier and more justified conclusions about the appropriateness of categorizing this compound as a candidate adjuvant for antibiotic therapy. In addition, the anti-*Acinetobacter* activity of oxazole-type compounds was recently proven by Trush *et al.,* 2020. In all tested derivatives included in this study, a high level of growth inhibition of MDR *A. baumannii* clinical isolates was also revealed [48].

**Table 1:** Antibacterial activity of AB15 against Gram-negative clinical bacterial isolates compared to the reference internal quality control strain, *Escherichia coli* (ATCC 25922)

Bacterial strain (ATCC/ID No.)	Clinical specimen	MIC AB15 (µM)	MIC AB15 (mg/L)	MIC GEN (μM)	MIC GEN (mg/L)
*Escherichia coli* (ATCC 25922)	Quality control	**31.25–62.5**	**7–14**	2.094	1
*Proteus mirabilis* (12/21)	Urine	**31.25–125**	**7–28**	> 33.5	> 16
*Klebsiella pneumoniae* (14/21)	Rectum	**31.25–125**	**7–28**	2.094	1
*Klebsiella pneumoniae* (15/21)	Rectum	**31.25–125**	**7–28**	2.094	1
*Enterobacter cloacae* (16/21)	Urine	**31.25–125**	**7–28**	> 33.5	> 16
*Acinetobacter baumannii* (59/16)	Tracheal aspirate	**31.25–62.5**	**7–14**	1.047	0.5
*Escherichia coli* (27/21)	Stool	**31.25–62.5**	**7–14**	4.188	2
*Acinetobacter baumannii* (20/21)	Urine	**15.63–62.5**	**3.5–14**	0.523	0.25
*Pseudomonas aeruginosa* (21/21)	Rectum	**500**	**112**	> 33.5	> 16
*Enterobacter cloacae* (23/21)	Urine	**31.25–62.5**	**7 –28**	> 33.5	> 16
*Escherichia coli* (24/21)	Urinal catheter	**31.25–62.5**	**7–28**	2.094	1
*Pseudomonas aeruginosa* (26/21)	Larynx	** > 500**	** > 112**	8.375	4
*Acinetobacter baumannii* (1/23)	Tracheal aspirate	**31.25**	**7**	> 33.5	> 16

ATCC, American Type Culture Collection; ID No., internal laboratory identification number; GEN, gentamicin; MIC, minimum inhibitory concentration. Determined by the microdilution broth method according to EUCAST recommendations. MIC was evaluated by visual inspection via the metabolic indicator Alamar Blue, and by spectrophotometric measurement. The results were read after 24 h of incubation at 37 °C.

### AB15 shows bactericidal action against Acinetobacter baumannii clinical isolate

As presented in Table S4 (Supplementary Information), only a slight (one-step) shift in the MIC of AB15 was registered in the reference strain, *A. baumannii* (ATCC 19609), evaluated after both 24 and 48 h. This only slight decrease in activity recognized after 48 h could indicate the bactericidal mode of the action of AB15. To determine the cidal activity of AB15, the microdilution method with a subsequent spread plate technique and the clinical isolate *A. baumannii* (20/21) were employed. After the exposure of *A. baumannii* to AB15 at a concentration level corresponding to fourfold MIC (250 µM), the percentage reduction in the number of CFU/mL corresponded to 99.983–99.984%, compared to the initial inoculum (Table S9, Supplementary Information). According to the mentioned criterion in the Materials and Methods section (Distinguishing between the bactericidal and static effect of AB15), AB15 appears to be a bactericidal compound. The cidal effect of a thiazole-based compound was also recently revealed by Haroun *et al.,* 2021, against MDR Gram-positive and Gram-negative bacteria [49]. The bactericidal or bacteriostatic effects of oxazole derivatives do not appear to have been extensively studied yet. Nevertheless, in a study conducted by Azzali *et al.,* 2020 [50], it was suggested that the activity of 2-aminooxazoles maintain similar cidal action compared to their 2-aminothiazole isosteres.

### Insight into the mechanism of the action of AB15

This study employed a macromolecular biosynthesis assay and a membrane depolarization assay for the recognition of bacterial biosynthesis pathway and/or cellular structures targeted by the compound AB15.

Macromolecular biosynthesis assay was performed according to study published by Nowakowska, *et al.,* 2013 with slight modifications [29]. This method is based on the exposure of bacteria to a tested compound and subsequent measurement of the percentage of [^3^H] radiolabeled precursors incorporated into newly synthesized biomacromolecules. After the measurement, data were compared with results obtained after exposing bacteria to commercial antibacterial drugs with known different mechanisms of action (inhibitors of DNA, RNA, peptidoglycan, and protein synthesis). When evaluating bacterial cytoplasmic membrane as a potential target of the tested compound, a voltage-sensitive dye, 3,3′-dipropylthiadicarbocyanine iodide (DiSC3(5)), CHX as the positive control and fluorometric measurement were employed.

**Fig. 4:**
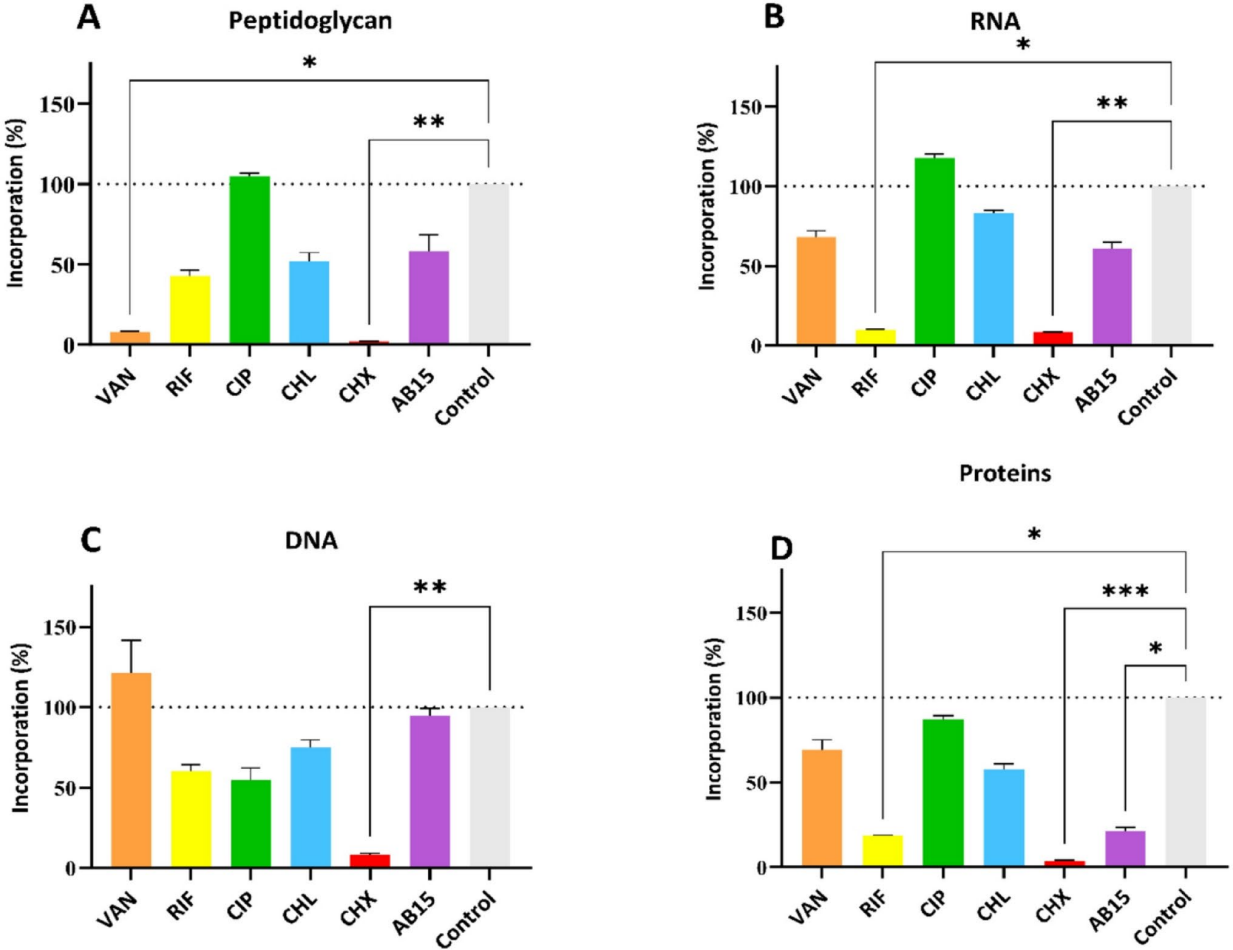
**A–D** Results of macromolecular biosynthesis assay. Inhibition of biosynthetic pathway is indicated by lower incorporation of radioactively labeled precursors. **A** [^3^H] *N*-acetylglucosamine (peptidoglycan synthesis), **B** [^3^H] uridine (RNA synthesis), **C** [^3^H] thymidine (DNA synthesis) and **D** [^3^H] leucine (protein synthesis) in methicillin-resistant *Staphylococcus aureus* (ATCC 43300) strain, treated for 2 h with 4 × MIC of VAN, RIF, CIP, CHL, CHX, and AB15. Results are expressed as the percentage of biomolecule incorporation related to untreated controls. The values shown are means of two independent experiments prepared in duplicates ± SEM. Significant reduction in biosynthetic pathway compared to control is indicated by *p*-value, where *p* < 0.05 was accepted as statistically significant (*p < 0.05; **p < 0.01; ***p < 0.001; ****p < 0.0001), determined by nonparametric one-way (ANOVA) test (Kruskal–Wallis test)

**Fig. 5:**
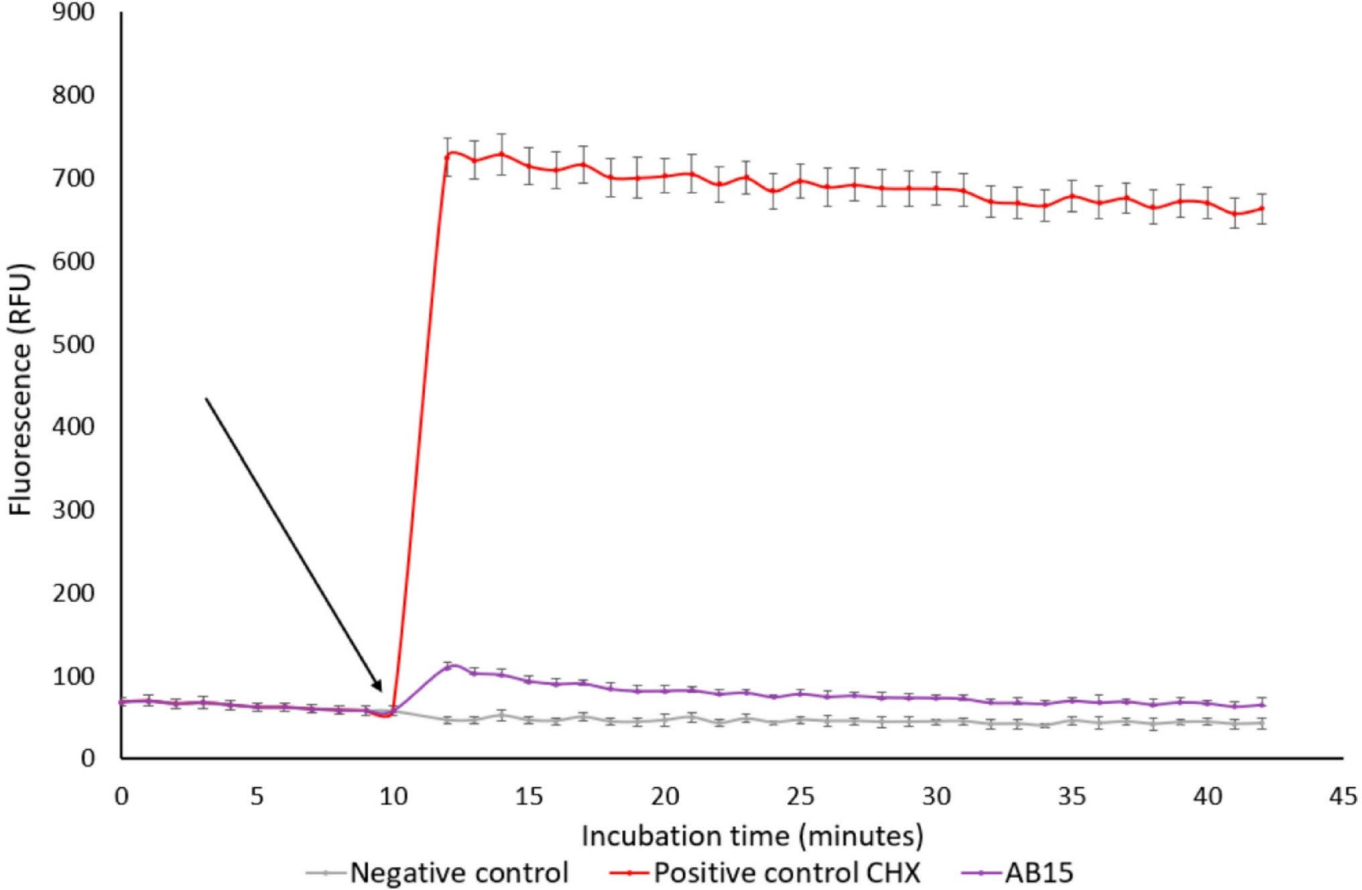
Membrane depolarization assay. Membrane potential was monitored using the fluorescence dye, DiSC3(5), (λ_Ex_ = 620 nm, λ_Em_ = 680 nm). Methicillin-resistant *Staphylococcus aureus* (ATCC 43300) was stained with 0.5 mM DiSC3(5). After 10 min of measurement (black arrow), AB15, positive control represented by chlorhexidine (CHX), in final concentrations corresponding to 4 × MIC were added. The graph depicts the mean of six replicates and the standard deviation

As shown in Fig. 4, the action of AB15 resulted in a statistically significant decrease in the incorporation of radiolabeled precursors participating in the protein synthesis pathway (Fig. 4D) of the reference bacterial strain MRSA (ATCC 43300) (used according to a protocol described in Nowakowska *et al.,* 2013) [29]. Therefore, it can be concluded that the protein synthesis pathway is primarily affected by the action of AB15. In addition, as a matter of fact, protein synthesis is recognized as a universally conserved macromolecular biosynthetic pathway in prokaryotic microorganisms, therefore, the same target can be expected in other bacterial species. In addition, acquired data from the membrane depolarization assay, shown in Fig. 5, revealed only a slight increase in bacterial membrane depolarization after treatment with 4 × MIC of AB15 (represented in purple), compared to the negative control (black). Thus, this finding suggests that the cytoplasmic membrane is not the target of AB15's effective activity.

### Focus on the *in vitro* cytotoxicity of AB15, and employment of invertebrate animal model, Galleria mellonella, for evaluating *in vivo* toxicity of AB15

**Fig. 6:**
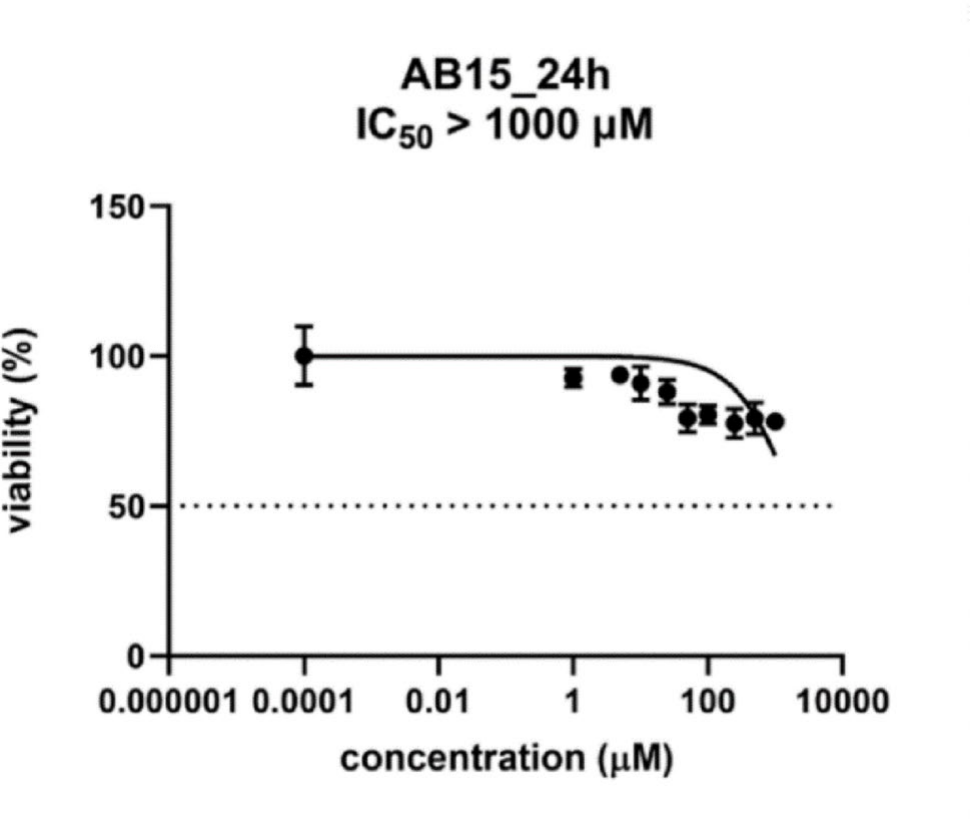
In vitro cytotoxicity of AB15 determined using the standard renal cell line HK-2 and expressed as the standard toxicological parameter IC_**50**_. IC_**50**_ was calculated by nonlinear regression from a semi-logarithmic plot of incubation concentrations versus the percentage of absorbance related to untreated controls using GraphPad Prism 9.0.0 (GraphPad Software, Inc., USA)

Newly synthesized compounds can be classified as candidate molecules for advanced studies and potential candidate molecules for antibiotic therapy fulfilling basic premises such as low or non-cytotoxicity expressed against eukaryotic cell lines. As was reported in our previous study [26], the cytotoxic potential recognized in AB15 against the employed standard hepatic cell line, human hepatoma cell line (HepG2), was negligible—the cytotoxicity parameter (IC_**50**_) corresponded to 664.1 µM [26]. Subsequently, to gain deeper insight into the cytotoxic and toxic potential of AB15, the standard renal cell line human kidney 2 (HK-2), and the alternative animal model, *Galleria mellonella,* were employed. Figure 6 demonstrates the cytotoxic effect of AB15 against the HK-2 cell line, with the IC_**50**_ > 1000 µM. This result indicates the lack of cytotoxic potential of AB15 against this employed cell line.

The compound efficacy* in vitro* can be expressed through the parameter known as selectivity index (SI). SI is calculated as the ratio of IC_**50**_/MIC. Compounds with SI above 10 are considered promising drug candidates since they present sufficient effectiveness and safety. As it was published in our previous study [26], after employing the HepG2 cell line, AB15 displayed a range of SI = 42.5–5.31 (calculated with MIC values for different strains). The employment of the HK-2 cell line led to a recognition of SI ranging from > 64 to > 16.

To assess the *in vivo* toxicity of AB15, an invertebrate model, *Galleria mellonella* larvae*,* was included. *Galleria mellonella* is a frequent substitute for animal models such as mice and rats in pathogenesis and toxicity studies. This model seems to be fully appropriate for distinguishing toxic and non-toxic chemicals. In addition, the 3Rs conditions (Replacement, Reduction, and Refinement), which are recognized as a framework for high-quality science in the academic sector, are fulfilled [51–53].

The larvae were divided into groups according to the final volume of administered compound per kg of the animal's body weight. The highest administered doses corresponded to 500 mg/kg of body weight, while the lowest doses had a final concentration of 5 mg/kg of body weight of larvae. Two ways of administration of the tested compound were employed, per oral administration by the force-feeding method, and injection into the hemocoel through the last left proleg, which mimics the intravenous route of administration in mammals and reflects systemic toxicity. As shown in Table S10 and Fig. S3 (Supplementary Information), the median lethal dose leading to the death of 50% of the animals (LD_50_) was not reached after the intra-hemocoel administration of AB15 in any group. Therefore, it can be concluded that LD_50_ of AB15 is higher than 500 mg/kg of body weight of the larvae. The highest mortality was registered in group one (500 mg/kg of body weight), corresponding to 12.5%. However, no mortality was registered in the other included groups (administered concentration ranged from 250 mg/kg to 5 mg/kg of body weight). Regarding toxicity after per oral administration, as shown in Table S11 and Fig. S4 (Supplementary Information), LD_50_ was not reached either. In addition, no larval deaths were recorded in any group at any inspection time interval. Therefore, it can be concluded that the recognized *in vivo* toxicity of AB15 after per oral administration corresponds to > 500 mg/kg. Based on these findings, AB15 could be categorized within the GHS (Globally Harmonized System) as class 4, which represents non/low toxic compounds [53].

After the administration of AB15 by both mentioned routes, no statistically significant effect on the survival of larvae was registered in a data analysis of all included groups via the pairwise Log-rank Mantel-Cox curve comparison test (Supplementary Information, Tables S12, S13).

In summary, the results from *in vitro* cytotoxicity, and *in vivo* toxicity evaluations indicate the promising ability of AB15, and this compound should be at least considered a possible adjuvant for antibiotic therapy.

### The mutual interaction of AB15 with selected, commercially available antibacterial drugs and its impact on antibacterial activity

In view of fact that AB15 showed promising activity against Gram-negative bacteria and showed low/non-toxic potential, it was further studied, what benefit the compound AB15 could have within the potential combination treatment strategy. Ideally, within antibiotic combination therapy, greater antibacterial activity of drugs in combination should be registered compared to the antibacterial action of drugs alone. Furthermore, reduced toxicity and mitigated adverse effects should be registered. In addition, the combination of antibacterial drugs, especially those with different mechanisms of action, reduces the risk of resistance development and spreading, which could alleviate the current advancing AMR crisis. To determine the mutual effect of AB15 with commercially available drugs, six different, preferentially selected, antibiotics were employed in this study, namely, CIP, GEN, TGC, SXT, CHL and CST. For preliminary insight, the bacterial strain *E. coli* (ATCC 25922) was employed. According to FICI values gathered in Tables S14–S19 (Supplementary Information), an additive effect was recognized at specific concentration ratios of AB15 in combination with GEN, CST, and CHL. The values of several FICI in combinations mentioned above were recognized to be very close to the categorization of synergy. For example, at some concentration ratios of AB15 + GEN, the FICI values corresponded to 0.516 and 0.531, respectively (the criterion to categorize the mutual interaction of two compounds as synergetic is FICI ≤ 0.5). Indifferent effect, revealed at other concentration ratios, indicates that the actions of compounds in combination are not mutually influenced. Finally, in all combinations of AB15 with selected antibiotic drugs at all tested concentration ratios, no antagonistic effect was recognized. Therefore, AB15 can be considered a valid candidate adjuvant molecule for supporting existing selected antibiotics.

**Fig. 7:**
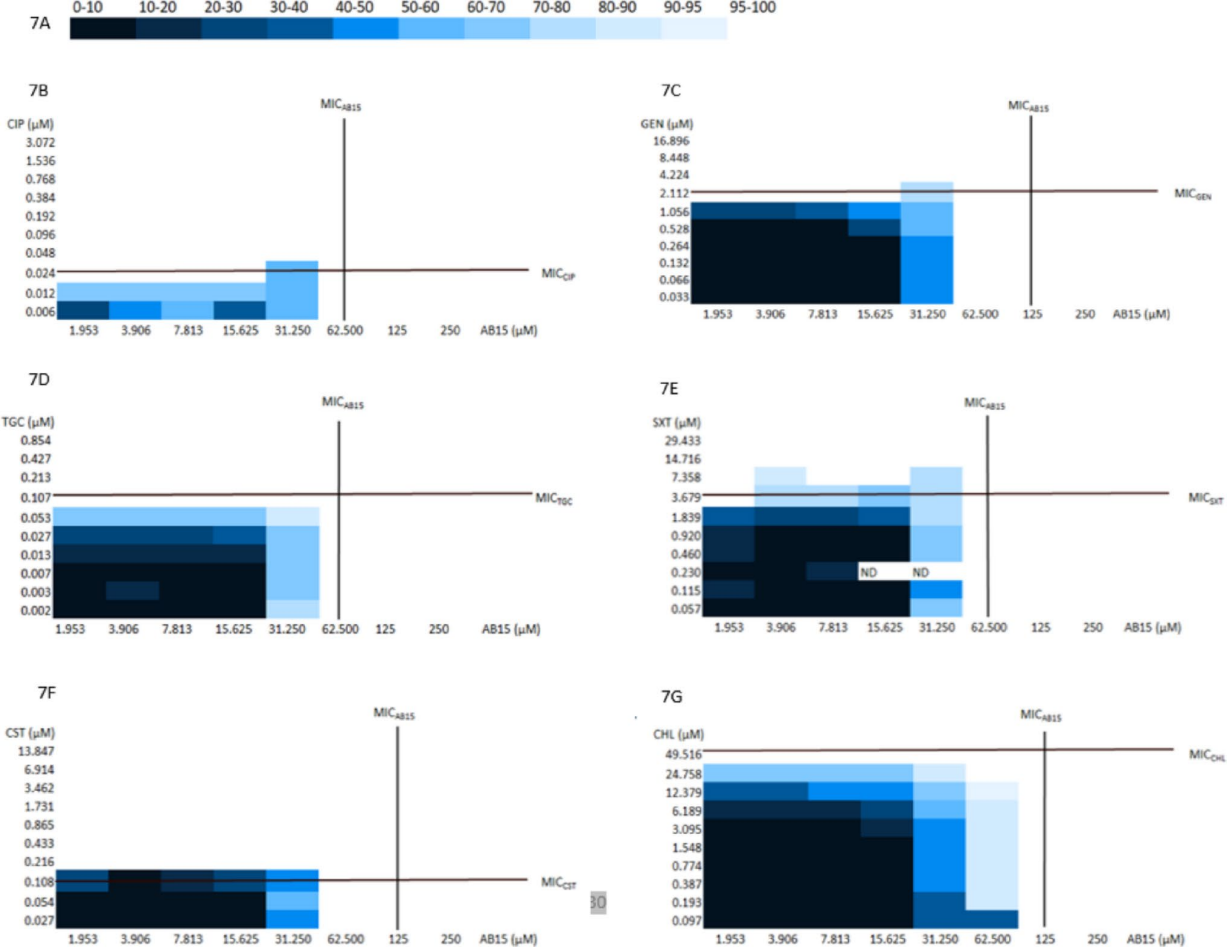
**A–G** Two-color heat maps of checkerboard MIC assays. The percentage of growth inhibition (compared to positive growth control) of bacterial strain, *Escherichia coli* (ATCC 25922), after 20 h of exposition at 37 °C to AB15 in combination with selected antibiotic drugs, expressed by color spectrum. **A** color spectrum**, B** AB15 in combination with ciprofloxacin (CIP), **C** gentamicin (GEN), **D** tigecycline (TGC), **E** trimethoprim-sulfamethoxazole (SXT), **F** colistin (CST) and **G** chloramphenicol (CHL). The black horizontal (selected antibacterial drug) and vertical (AB15) lines denote the MIC of each compound alone

For better clarity, the results of checkerboard assays are also presented in form of heat maps (Fig. 7B–G). Each figure illustrates the percentual growth inhibition of the reference bacterial strain *E. coli* after being exposed to AB15 in combination with selected antibiotic drugs. The percentage of growth inhibition is illustrated using a color scheme (Fig. 7A), where the darkest shade represents the lowest inhibition and, therefore, the highest growth of the employed bacterial strain, while the lightest shade indicates the highest achieved inhibition and, consequently, the lowest growth of *Escherichia coli.* As was seen within the combination AB15 + CIP and AB15 + CHL (Fig. 7B and G), there was no distinct contribution of AB15 to the activity of these two commercial drugs. Nevertheless, the AB15 + GEN, and AB15 + CST combinations led to the increased AB15 activity (Fig. 7C and F). The combination of AB15 + SXT appeared to be disadvantageous at some concentration ratios (Fig. 7E).

### AB15 has an additive effect with colistin against the MDR Acinetobacter baumannii strain

Our study explored the activity of AB15 against the most feared pathogen with regard to its multidrug resistance and limited possibilities of available effective antibiotic treatment, the clinical bacterial isolate, MDR *A. baumannii* (20/21)*.* CST is considered an antibacterial drug of last resort, primarily reserved for the treatment of serious infections caused by MDR Gram-negative bacterial strains. Its mechanism of action involves targeting lipopolysaccharides in the outer membrane of these bacteria. However, in recent years, a rapidly increasing number of reports describing emerging cases of bacterial resistance to CST have been published [54–56].

Studies focused on the use of CST within combination therapy have been widely discussed in recent years. For example, in a study published by Brennan-Kohn et al*.,* 2018, the authors focused on various combinations of antibiotics with CST. In 18 out of 19 combinations, a synergistic effect against more than two bacterial clinical isolates from the *Enterobacteriaceae* family was registered [57]. Furthermore, in a study conducted by Almutairi *et al*., 2022, the synergistic interaction of two drug combinations of CST with VAN, aztreonam, ceftazidime, and imipenem was revealed against clinical isolates of colistin-resistant *A. baumannii* [58].

CST seems to be the last relevant antibiotic, which is available to treat severe infections caused by MDR *A. baumannii.* Clinicians often choose polymyxins in combination with other antibiotics. Nevertheless, the relevance of only some of them can be proven by published studies. For example, Khalil *et al.,* 2019, demonstrated the beneficial action of CST in combination with trimethoprim-sulfamethoxazole [59]. On the other hand, some studies have pointed out that the efficacy of CST monotherapy compared to combination therapy with CST of infections caused by *A. baumannii* is comparable [60–62]. It should be mentioned that the limitations of CST monotherapy lie in its undesirable neurotoxicity [63] and nephrotoxicity [64].

**Fig. 8:**
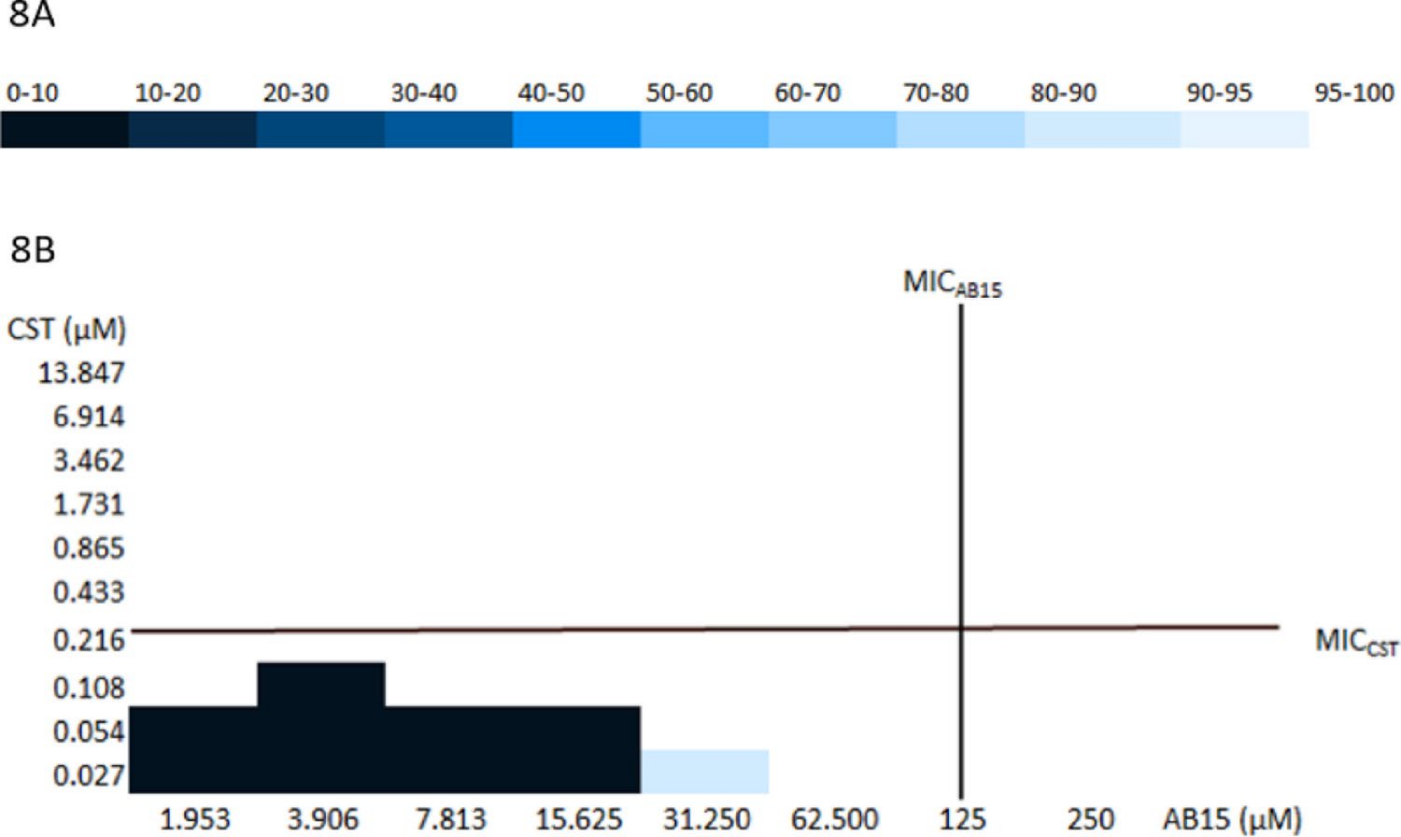
**A**,** B** Two-color heat map of the checkerboard MIC assay. The percentage of growth inhibition (compared to the positive growth control) of the bacterial strain, *Acinetobacter baumannii* (20/21) after 20 h of exposure at 37 °C to AB15 in combination with colistin (CST), expressed by the color spectrum. The minimum inhibitory concentration (MIC) of compounds alone corresponds to: MIC_CST_ = 0.216 µM, MIC_AB15_ = 125 µM. **A** color spectrum, **B** AB15 in combination with colistin (CST)

The additive effect of the AB15 + CST combination revealed against *E. coli* was subsequently evaluated against the clinical isolate MDR *A. baumannii* (20/21). As illustrated in Table 2, the synergistic potential at concentration ratios corresponding to 0.054 µM CST + 31.25 µM AB15 was revealed. The concentration ratio 0.108 CST µM + 7.813 µM AB15 was recognized as being very close to meeting the FICI criterion for classifying the interaction as synergistic (FICI = 0.563). Additivity was revealed at four other concentration ratios. In addition, no antagonistic interaction was registered within the evaluated ratios. concentration ratios. In addition, no antagonistic interaction was registered within the evaluated ratios. Similarly to the checkerboard studies including *E. coli*, the antibacterial activity of the CST and AB15 in combination is graphically illustrated in a heat map (see Fig. 8A, B). Generally, the antibacterial action of CST is potentiated by the presence of AB15, and vice versa, the presence of AB15 is potentiated by CST.

**Table 2:** Total fractional inhibitory concentration indexes (FICI) determined by checkerboard assay with a combination of colistin (CST) and AB15. The clinical bacterial isolate, *Acinetobacter baumannii* (20/21) was included in the assay. The minimum inhibitory concentration (MIC) of the compounds alone was: MIC_CST_ = 0.216 µM, MIC_AB15_ = 125 µM

Combination of compounds (colistin: AB15) vs *Acinetobacter baumannii*					
Concentration ratio CST: AB15 (µM)	Concentration ratio(meena)CST: AB15(meena)(mg/L)	FIC_CST_	FIC_AB15_	FICI	Effect
0.027: 62.500	0.031: 14.000	0.125	0.500	0.625	**Additivity**
0.054: 31.250	0.063: 7.000	0.250	0.250	0.500	**Synergy**
0.108: 31.250	0.125: 7.000	0.500	0.250	0.750	**Additivity**
0.108: 15.625	0.125: 3.500	0.500	0.125	0.625	**Additivity**
0.108: 7.813	0.125: 1.750	0.500	0.063	0.563	**Additivity**
0.216: 3.906	0.250: 0.875	1.000	0.031	1.031	Indifference
0.216: 1.953	0.250: 0.438	1.000	0.016	1.016	Indifference

FIC, Fractional Inhibitory Concentration, FICI, Fractional Inhibitory Concentration Index, FIC(CST, AB15) = MIC of the combination/MIC_CST, AB15_ alone, FICI = FIC_CST_ + FIC_AB_. The effect was interpreted as follows: synergy when FICI ≤ 0.5, an additive effect when 0.5 < FICI ≤ 1, an indifferent effect when 1 < FICI ≤ 4, and an antagonistic effect when FICI > 4.

### AB15 potentiates the antibiofilm effect of CST by suppressing the dissemination of bacteria from biofilm consortia formed by a clinical isolate of Acinetobacter baumannii

The ability to form biofilms strongly contributes to the *A. baumannii* broad spectrum of virulence and resistance factors. As mentioned above, *A. baumannii* thrives in hospital environments and is capable of creating biofilm consortia on numerous medical devices and surfaces. Biofilm formation promotes the resistance of *A. baumannii* cells and plays a crucial role in its pathogenesis. *A. baumannii* is the causative agent of many life-threatening infections, such as catheter-related infections, meningitis, urinary tract infections, ventilator-associated pneumonia, etc., whose treatment is highly challenging [65–67].

With these facts in mind, it was entirely appropriate within this study to focus our attention on the anti-biofilm activity of AB15. Concerning the promising results achieved in the study of AB15 as a potential adjuvant compound in combination with the last-resort drug CST, the concentration ratio of 0.108 µM CST + 7.813 µM AB15 was selected for the determination of anti-biofilm activity.

Clinical isolate of *A. baumannii* (20/21) was characterized as a strong biofilm producer based on a categorization provided in 2007 by Stepanović *et al*. [68].

*A. baumannii* biofilm consortia were formed *in vitro* and were subsequently exposed to AB15 and CST, both acting alone and in combination. For determination of the anti-biofilm activity of these antibacterial-acting compounds in combination, the multiples of concentrations ranging from 1 × to 128 × MIC corresponding to a concentration ratio of 7.813 µM (AB15) + 0.108 µM (CST) were employed. To describe the anti-biofilm activity, two parameters defined in the experimental part of this study, MBDC and MBEC, were determined using the microtiter plate biofilm assay. In this work, MBDC expresses the concentration of compound(s) leading to 95% reduction in metabolic activity of the planktonic cells released from biofilm consortia, compared to the activity of released cells without any exposure to the tested compound(s). An alternative approach to evaluate the MBEC parameter, the metabolic indicator, Alamar Blue, and a step of metabolic activity restoration of persistent cells was included in this study [69, 70]. MBEC expresses the concentration of compound(s) leading to 95% inhibition in metabolic activity of the bacterial cells embedded in biofilm consortia, after restoration of metabolic activity of persistent cells, compared to the positive growth control, represented by biofilm-forming bacteria unexposed to any tested compound(s). As shown in Table 3, AB15 seems to have greater intrinsic anti-biofilm activity compared to CST. The MBDC and likewise MBEC values corresponded only to a 2–4 × higher concentration that was needed for the metabolic inhibition and growth of bacterial cells of *A. baumannii* present in planktonic form (MIC value). In CST, the MBDC corresponded to a 64–256 × higher concentration necessary for the metabolic inhibition of planktonic cells. Focusing on MBEC, only 2–4 × multiple AB15 concentration compared to MIC was necessary for the 95–100% metabolic inhibition of biofilm-forming *A. baumannii*. In CST, MBEC corresponded to a 16–64 × multiplicity of MIC. The MBDC and MBEC of the CST + AB15 combination corresponded equally to a ratio of 1.728 µM CST + 125 µM AB15. The concentrations of the compounds present in this combination corresponded to a 16 × multiplicity of CST + AB15 concentrations in combination needed for the metabolic inhibition of bacteria in planktonic form. Based on these results, it can be concluded that AB15 undoubtedly exhibits beneficial attributes to be a potential partner compound to CST. Considering the action of CST against biofilms formed by *A. baumannii*, the appropriate concentration ratio of CST with AB15 allows to lower the concentration of this drug with clear adverse effects from 27.695 µM (CST acting alone) to a concentration of 1.728 µM (in combination with AB15).

**Table 3:** The minimum biofilm inhibitory concentration of colistin (CST), AB15, and the AB15 + CST combination against biofilm-forming *A. baumannii* (20/21) cells

	MIC (µM)	MBDC (µM)	MBEC (µM)	Multiplicity of MIC to MBDC	Multiplicity of MIC to MBEC
CST	0.108–0.216	13.847–27.695	3.462–6.924	64–256	16–64
AB15	31.25–62.5	125	125	2–4	2–4
*CST + AB15	0.108 + 7.813	1.728 + 125.000	1.728 + 125.000	16	16

*Concentration ratio with recognized mutual additive action of colistin (CST) and AB15, selected for the anti-biofilm activity study

MIC–the concentration of compound(s) leading to the 95–100% reduction of metabolic activity of bacteria in planktonic form compared to positive growth control (bacteria unexposed to tested compound(s)); MBDC–the concentration of compound(s) leading to the 95–100% reduction of metabolic activity of planktonic bacteria released from biofilm consortium; MBEC—the concentration of compound(s) leading to the 95–100% reduction of metabolic activity of bacteria present in biofilm consortium.

**Fig. 9:**
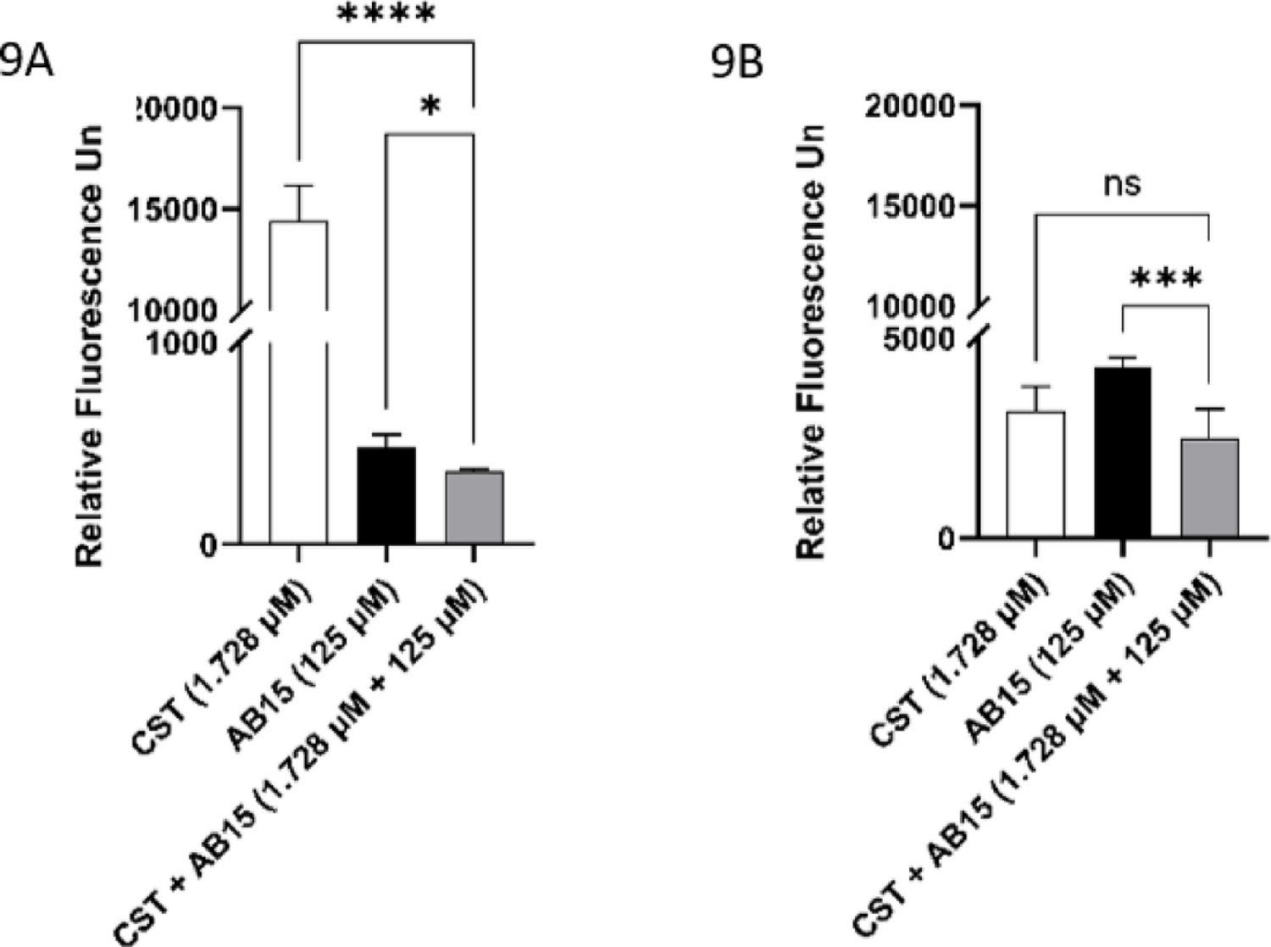
**A, B** A comparison of the effectiveness of colistin (CST, 1.728 µM), AB15 (125 µM), both acting individually and in combination of CST + AB15 at a concentration ratio of 1.728 µM CST + 125 µM AB15, in **A** suppressing the dissemination of bacterial cells from biofilms, and **B** targeting biofilm-embedded cells. The biofilm-forming *Acinetobacter baumannii* (20/21) was exposed to CST, AB15, and CST + AB15 for 24 h at 37°C. The metabolic fluorescent indicator, Alamar Blue (λ_Ex_ = 528 nm and λ_Em_ = 585 nm), was employed to determine the anti-biofilm activity. One-way analysis of variance (ANOVA, Kruskal–Wallis test) was performed to make a direct group–group comparison, and a *p*-value < 0.05 was accepted as statistically significant. The data are presented as the mean ± standard error of the mean. *p < 0.05; **p < 0.01; ***p < 0.001; **** *p* < 0.0001. Data was processed using GraphPad Prism 9.0.0 (GraphPad Software, Inc., USA)

The mutual contribution to the anti-biofilm action of AB15 + CST can also be deduced from a comparative analysis of the bacterial metabolic activity achieved and measured after exposure to the tested compound(s). The fluorescence signal resulting from the conversion of the metabolic indicator, Alamar Blue, was measured. As shown in Fig. 9A, the combination of CST + AB15, compared to the action of either CST or AB15 alone, leads to a statistically significant contribution within the anti-biofilm action reflected by the capacity of the biofilm consortium to release metabolically active bacterial cells (MBDC). After focusing on CST alone, AB15 alone, and CST + AB15 in action against bacterial cells, which remained participants of biofilm communities during the whole time of the exposure (MBEC parameter), the evident benefit from the CST + AB15 combination was recognized only for AB15 (Fig. 9B). However, within the evaluation of anti-biofilm activity, the MBEC parameter should not be considered or prioritized as the sole criterion. The process of bacterial detachment from biofilm consortia is recognized as an integral part of the life cycle of biofilm-forming communities. These biofilm-dispersed cells stay behind the dissemination of infection through the host environment and the formation of new infectious biofilm deposits [71]. Therefore, recognizing the potential to limit the spread of infectious agents from biofilm deposits should be considered a valuable attribute of antibacterial compounds, whether acting alone or in combination.

### Demonstration of the benefit of the combination of CST and AB15 related to potential cytotoxicity to human red blood cells

CST has cationic detergent activity and binds to lipopolysaccharides in the bacterial outer membrane, displacing calcium and magnesium, which leads to the disruption of the lipid components of the cell membrane. Further, this drug is administered intravenously to critically ill patients [72]. Therefore, our attention has been directed to the determination of hemolytic activity of CST alone, and CST and AB15 in combination.

CST was re-emerged as a last resort antimicrobial drug, despite its reported adverse effects, namely nephrotoxicity [64] and neurotoxicity [63], which are often dose-dependent and reversible on discontinuation of treatment. As it was shown in the study by Westenfelder, *et al*., 2022, increased levels of hemoglobin in the circulation, caused by hemolysis, contribute to hem-induced proximal tubular acute kidney failure [73]. In conclusion, any increase in hemolytic activity of CST and AB15 in combination should be considered an undesirable manifestation of the biological activity.

For the determination of the cytotoxic (hemolytic) potential of the CST alone and CST and AB15 in combination, an ex vivo human red blood cell (RBC) hemolysis assay was employed. Fresh human RBCs were exposed to CST and CST in combination with AB15 in final concentrations corresponding to concentrations with anti-biofilm effect established in the previous experiment (256 × MIC_CST_ and 16 × MIC_CST+AB15_). After incubation and centrifugation steps, hemoglobin released from RBC was monitored by measuring the absorbance at 405 nm.

**Fig. 10:**
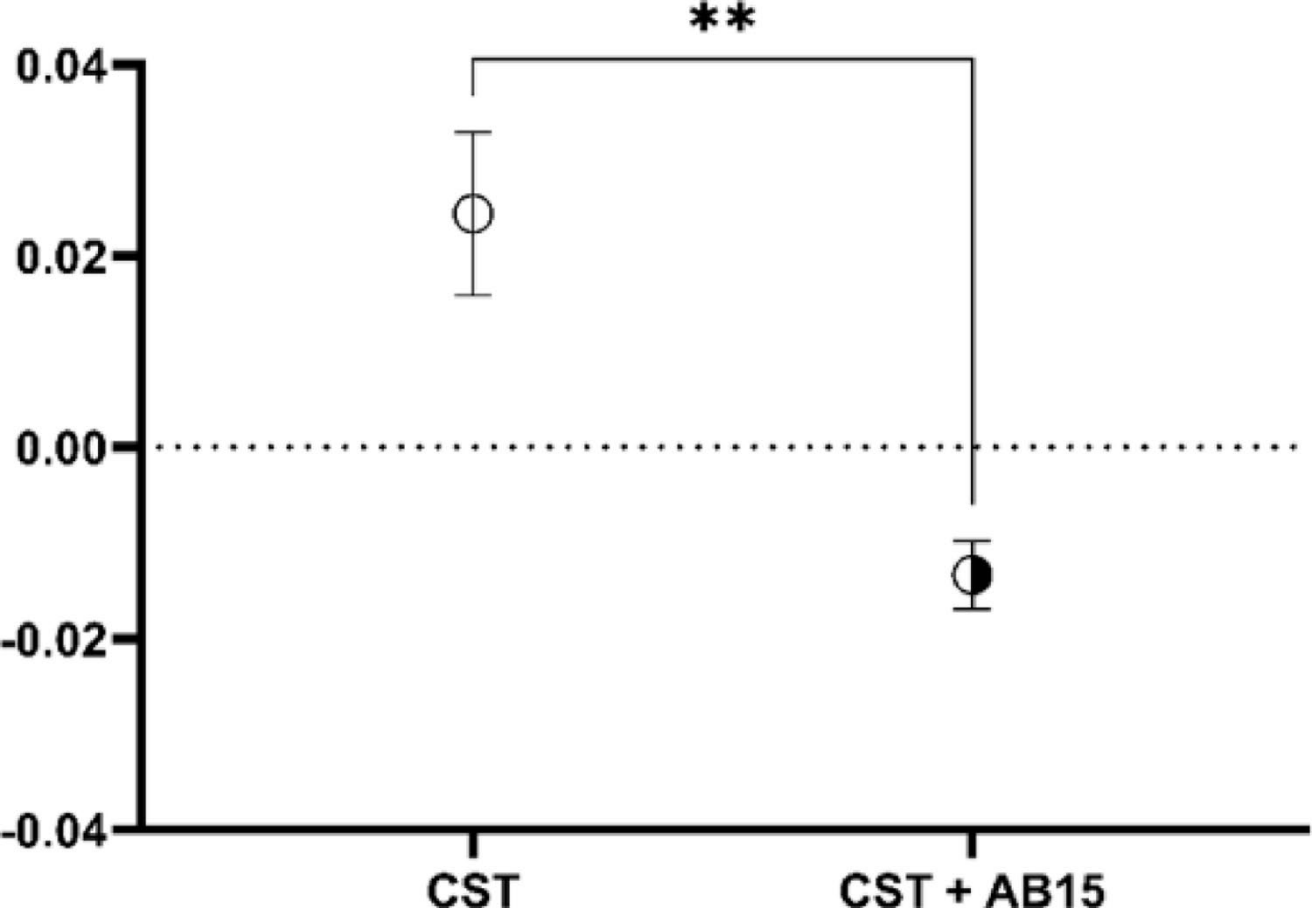
Evaluation of hemolytic activity of colistin (CST) alone in final concentrations corresponding to 256 × MIC_CST_ (55.3 mM), and CST in combination with AB15 in final concentration corresponding to 16 × of MIC_CST+AB15_ (1.728 mM CST + 125 mM AB15). The amount of hemoglobin released into the supernatant was measured at 405 nm. Data were subtracted from the negative control (unexposed RBC) and provided using a mean of hexaplicates ± SEM. Statistical significance is indicated by *p*-value, where *p* < 0.05 was accepted as statistically significant (**p < 0.01), determined by nonparametric one-way ANOVA test (Kruskal–Wallis test)

As shown in Fig. 10, the CST and AB15 in combination show statistically significant lower cytotoxic activity towards human RBC, compared to CST alone. Thus, this combination suggests better management of CST for the treatment of infectious diseases and a subsequent decrease in the risk of CST-associated nephrotoxicity.

## Conclusion

In this study, we focused on gaining extensive and comprehensive insight into the antibacterial action and (cyto-)toxic potential of a recently published 2-chloro-*N*-(oxazol-2-yl)isonicotinamide (AB15) [26].

Our extensive study, including bacterial clinical isolates with determined MDR profiles, revealed a promising activity against a member of ESKAPE, *A. baumannii*, with the MIC of 15.625–62.5 µM. The activity against other included Gram-negative members of ESKAPE, namely *E. coli*, *Klebsiella pneumoniae, Proteus mirabilis,* and *Enterobacter cloacae*, ranged from 31.25 to 125 µM. The activity against *Pseudomonas aeruginosa* clinical isolate was negligible (500 µM, > 500 µM). AB15 had low to no cytotoxicity *in vitro* (HepG2, HK-2 cell lines) and *in vivo* (larvae of *G. mellonella* after per oral and intra-hemocoel administration).

Regarding mechanisms of action, AB15 displays a bactericidal effect against a clinical isolate *A. baumannii* and targets dominantly bacterial protein synthesis. The exact molecular target for AB15 inhibiting protein synthesis remains to be elucidated. To assess *in vitro* cytotoxicity and *in vivo* toxicity, a human kidney epithelial cell line HK-2, and an alternative animal model, *Galleria mellonella,* were included in this study. The employment of the HK-2 cell line led to the recognition of SI ranging from > 64 to > 16. The recognized *in vivo* toxicity parameter LD_50_ of AB15 after intra-hemocoel and per oral administration into the *G. mellonella* model corresponded to a value > 500 mg/kg of body weight that allows us, according to GHS system of categorization, to include AB15 as a non/low toxic compound. Our investigation of the mutual effect of AB15 with selected commercially available antibiotics (CIP, GEN, TIG, SXT, CHL, CST) against *E. coli*, revealed that at no concentration ratios, the antagonistic effect was achieved. An additive effect was registered in a combination of AB15 with GEN, CST, and CHL. A highly beneficial, synergic, and additive effect was revealed for a combination of AB15 with CST against *A. baumannii* clinical isolate. Finally, it was demonstrated that AB15 in combination with CST contributes to antibiofilm activity expressed by reduction of bacterial dissemination from biofilms formed by MDR clinical isolate *A. baumannii*. The biocompatibility towards human erythrocytes is improved for AB15 + CST combination, compared to CST acting individually at an equivalent antibiofilm-effective concentration. The relevance of AB15 as a potential adjuvant of CST can be strongly enhanced by the finding that both compounds, CST and AB15, show different mechanisms of action. In general, combining compounds with diverse mechanisms of action reduces the risk of the development and spreading the AMR.

To conclude, based on the observed antibacterial and biological attributes as well as for its favorable physico-chemical properties, AB15 can be considered a valid candidate adjuvant molecule to support existing selected antibiotics.

## Supplementary Information

Below is the link to the electronic supplementary material.

Supplementary Material 1

Supplementary file1 (DOCX 14 kb)

## Data Availability

No datasets were generated or analysed during the current study.
