# Supplementary material for: A new bactericidal chlorinated derivative containing 2-aminooxazole potentiates antibacterial action of colistin against multidrug-resistant acinetobacter baumannii
Source: Med Microbiol Immunol. 2025 Sep 19;214(1):44. doi: 10.1007/s00430-025-00854-y (PMC12449380; doi:10.1007/s00430-025-00854-y)
Supplement: Supplementary file 1 — Supplementary Material 1 [file 430_2025_854_MOESM1_ESM.docx]

A NEW BACTERICIDAL CHLORINATED DERIVATIVE CONTAINING 2-AMINOOXAZOLE POTENTIATES ANTIBACTERIAL

ACTION OF COLISTIN AGAINST MULTIDRUG-RESISTANT

*ACINETOBACTER BAUMANNII*

SUPPLEMENTARY INFORMATION

Adéla Diepoltová^1^, Daria Elzbieta Nawrot^1^, Ondřej Janďourek^1^, Martin Juhás^1,2^, Pavel Bárta^1^, Pavlína Vávrová^1^, Vinod Sukanth Kumar Pallabothula^1^, Paulína Dudášová-Hatoková^1^, Marcela Vejsová^1^, Barbora Voxová^1^, Jan Österreicher^1^, Petra Štěrbová-Kovaříková^1^, Petr Nachtigal^1^, Jan Zitko^1^, Klára Konečná^1^*****

1 Charles University, Faculty of Pharmacy in Hradec Králové, Akademika Heyrovského 1203, 500 03

Hradec Králové, Czech Republic

2 University of Hradec Králové, Department of Chemistry, Faculty of Science, Rokitanského 62, 500 03 Hradec Králové III, Czech Republic

***** Correspondence to Klára Konečná, [konecna@faf.cuni.cz,](mailto:konecna@faf.cuni.cz) Tel: + (420)495067366, ORCID:0000-0001-5670- 7767

**MATERIALS AND METHODS**

^1^H- and ^13^C-NMR spectra were recorded at ambient temperature using Jeol JNM-ECZ600R (Jeol Ltd., Tokyo, Japan) spectrometer operating at 600 MHz for ^1^H and 151 MHz for ^13^C. Chemical shifts were reported as values in ppm and were indirectly referenced to tetramethylsilane (TMS) *via* the solvent signal (2.50 for ^1^H and 39.7 for ^13^C in DMSO). Coupling constants *J* are given in Hz. The infrared spectrum was recorded on FT-IR Nicolet 6700 spectrometer (Thermo Scientific,Waltham, MA, USA) using the attenuated total reflectance (ATR) method on a germanium crystal. The purity was measured using a Nexera® UHPLC system (Shimadzu, Kyoto, Japan) coupled with a PDA detector (SPD-M20A) on the Ascentis® C18 (100 3 mm, 3 m, Supelco®) column using an acetonitrile/0.5% formic acid mobile phase mixture in isocratic mode. Data were processed using LabSolutions software (v. 5.92, Shimadzu, Kyoto, Japan). The stock solution of compound AB15 (0.5 mg/mL) was prepared by dissolving the appropriate amount in methanol. Working solution was prepared by diluting the stock solution with the acetonitrile/water mixture (1:1, v/v) to a concentration of 50 µg/mL. The PDA detector acquired spectra from 190 to 380 nm, and a wavelength of 254 nm was employed for purity evaluation.

The HRMS identification was performed using the Q-Exactive Focus (Thermo Scientific, San José, CA, USA) with HESI, and the data were processed with Xcalibur software (Thermo Scientific). Heated-electrospray ionization II interface (HESI-II) in positive ion mode was used with the following settings: spray voltage, 0.5–5 kV; S-lens RF level, +50 V; capillary temperature, 350°C; auxiliary gas heater temperature off; sheet and auxiliary gas flow, 5 and 2 arbitrary units, respectively. Data were acquired in full-scan MS mode (FullMS) at resolution (m/Dm) ≈ 70 000 with the accuracy of measuring –1.71 ppm with quadrupole filter mass range 90–450 m/z.

**Results of analytical characterization of compound AB15 by nuclear magnetic resonance spectroscopy (NMR), infrared spectroscopy (IR), high-resolution mass spectrometry (HRMS), and high-performance liquid chromatography (HPLC)**

2-Chloro-*N*-(oxazol-2-yl)isonicotinamide (**AB15**).

Light brown solid. Yield 25 %. mp = 158–162 °C.

IR (ATR-Ge, cm^−1^) 3205, 1615 (υNHCO), 1591, 1576, 1541, 1463.

^1^H NMR (600 MHz, DMSO-*d_6_*): δ 12.24 (s, 1H), 8.60 (d, *J* = 5.0 Hz, 1H), 7.97 (d, *J* = 1.3 Hz, 1H), 7.92 (d, *J* = 1.0 Hz, 1H), 7.89 (dd, *J* = 5.0, 1.3 Hz, 1H), 7.27 (d, *J* = 1.0 Hz, 1H).

^13^C NMR (151 MHz, DMSO- *d_6_*): δ 150.89, 150.77, 122.68, 121.51.

HRMS (HESI+) m/z = 224.0217 (theoretical for [M+H]^+^ m/z = 224.0221, error –1.71 ppm). HPLC purity 99.4%.


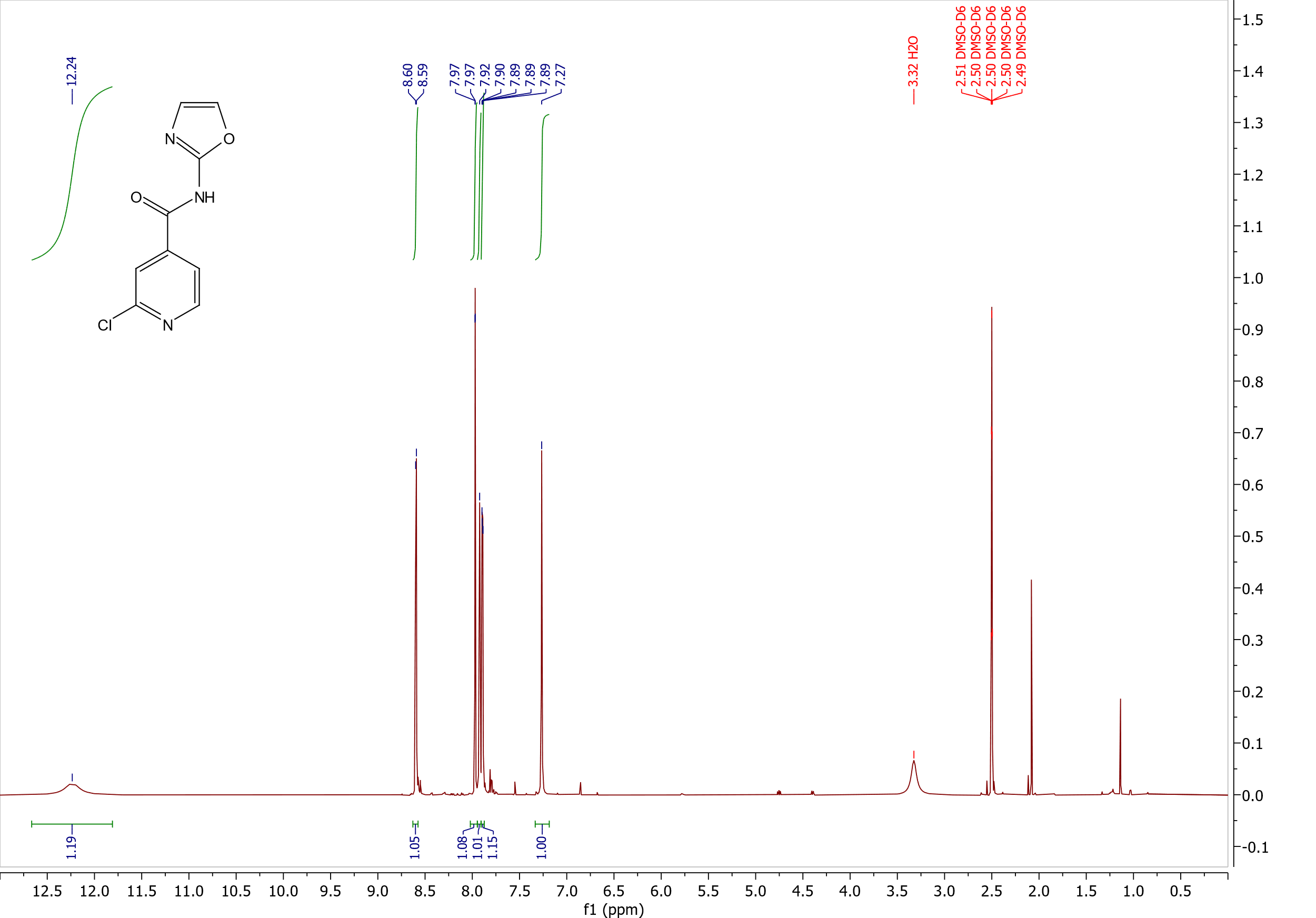


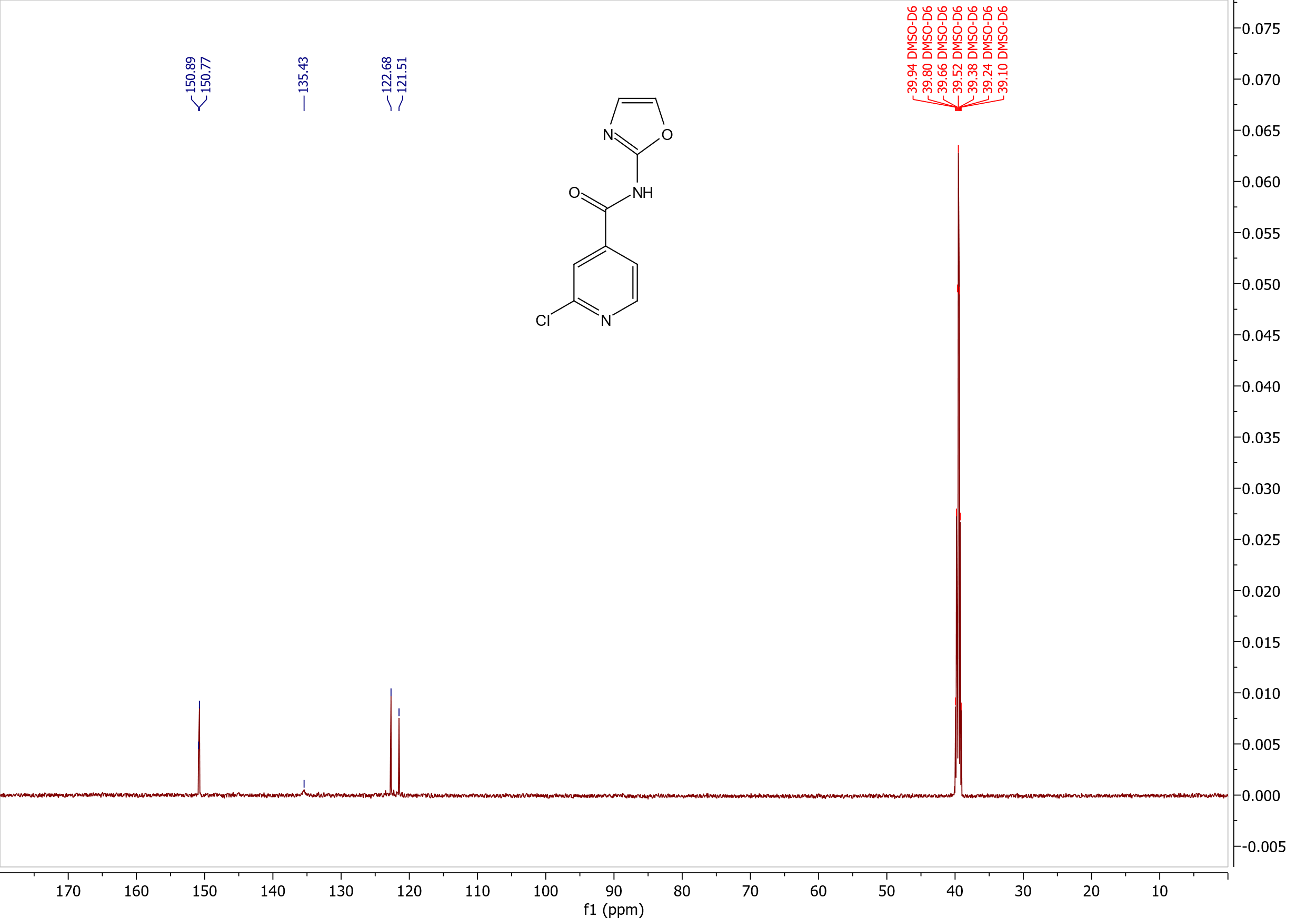


**Figure S1**. ^1^H NMR (top) and ^13^C NMR (bottom) spectra of compound AB15


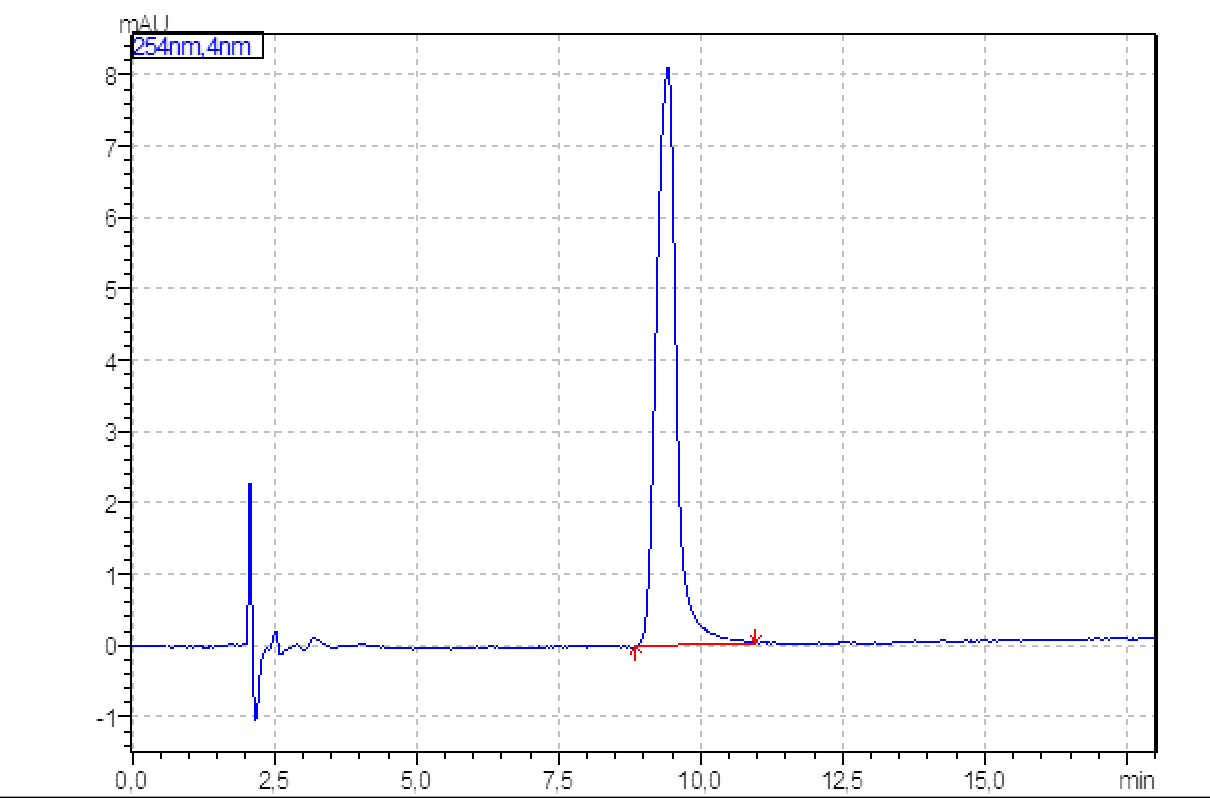


**Figure S2.** UHPLC chromatogram of compound AB15 (purity 99.4% at 254 nm)

**Table S1.** Antibacterial activity of internal quality standards (ciprofloxacin and gentamicin) against clinical bacterial isolates and reference internal quality control strains (*Staphylococcus aureus* (ATCC 29213) and *Escherichia coli* (ATCC 25922). The antibacterial activity was determined by the microdilution broth method according to EUCAST recommendations. The Minimum Inhibitory Concentration was evaluated by visual inspection *via* the metabolic indicator Alamar Blue, and by spectrophotometric measurement. The results were read after 24 h of incubation at 37 °C.

| Bacterial strain (ATCC/ID No.) | Clinical specimen | MIC CIP (µM) | MIC CIP (mg/L) | MIC GEN (µM) | MIC GEN (mg/L) |
| --- | --- | --- | --- | --- | --- |
| *Staphylococcus aureus subsp. aureus (ATCC 29213)* | Quality control | 0.77 | 0.256 | 2.09 | 1 |
| *Staphylococcus aureus subsp. aureus, methicillin-resistant (131/16)* | Throat swab | >6.181 | >2.048 | 1.05 | 0.5 |
| *Staphylococcus aureus, vancomycin-resistant (NIPH)* | ND | 0.77 | 0.256 | 4.19 | 2 |
| *Staphylococcus capitis (9/21)* | ND | 0.77 | 0.256 | 2.09 | 1 |
| *Staphylococcus haemolyticus (11/21)* | ND | >6.181 | >2.048 | >33.5 | >16 |
| *Escherichia coli* (ATCC 25922) | Quality control | 0.012 | 0.004 | 2.094 | 1 |
| *Proteus mirabilis (*12/21) | Urine | > 6.181 | > 2.048 | > 33.5 | > 16 |
| *Klebsiella pneumoniae* (14/21) | Rectum | 3.090 | > 2,048 | 2.094 | 1 |
| *Klebsiella pneumoniae* (15/21) | Rectum | 3.090 | 1.024 | 2.094 | 1 |
| *Enterobacter cloacae* (16/21) | Urine | > 6.181 | > 2.048 | > 33.5 | > 16 |
| *Acinetobacter baumannii* (59/16) | Tracheal aspirate | 0.193 | 0.064 | 1.047 | 0.5 |
| *Escherichia coli* (27/21) | Stool | > 6.181 | > 2.048 | 4.188 | 2 |
| *Acinetobacter baumannii* (20/21) | Urine | 0.386 | 0.128 | 0.523 | 0.25 |
| *Pseudomonas aeruginosa* (21/21) | Rectum | > 6.181 | > 2.048 | > 33.5 | > 16 |
| *Enterobacter cloacae* (23/21) | Urine | 6.181 | 2.048 | > 33.5 | > 16 |
| *Escherichia coli* (24/21) | Urinal catheter | 0.048 | 0.016 | 2.094 | 1 |
| *Pseudomonas aeruginosa* (26/21) | Larynx | > 6.181 | > 2.048 | 8.375 | 4 |
| *Acinetobacter baumannii* (1/23) | Tracheal aspirate | > 6.181 | > 2.048 | > 33.5 | > 16 |

*ATCC – American Type Culture Collection, CIP – ciprofloxacin, GEN – gentamicin, ID No. – internal laboratory identification number; NIPH – National Institute of Public Health*

**Table S2.** Selected medicinal chemistry, physico-chemical, and pharmacokinetic parameters of AB15

| **Basic medicinal chemistry rules** | | | | | |
| --- | --- | --- | --- | --- | --- |
| Parameter | Value | Lipinski | Veber | Muegge | Oprea |
| MW | **223.62*** | **≤500** | n.d. | **200–600** | **300–400** |
| H-bond donors | **1*** | **≤5** | n.d. | **≤5** | **≤3** |
| H-bond acceptors | **4*** | **≤10** | n.d. | **≤10** | **≤6** |
| Predicted logP (consensus) | **1.32*** | **≤5** | n.d. | **-2 to 5** | **≤3.5** |
| Number of rotatable bonds | **3*** | n.d. | **≤10** | **≤15** | n.d. |
| TPSA (Å²) | **68.02*** | n.d. | **≤140** | **≤150** | n.d. |
| Ring count | **2** | n.d. | n.d. | **≤7** | n.d. |
| Compliant to medico-chemical rules | **Yes** | **Yes** | **Yes** | **Yes** | **Yes** |
| **ADMET prediction by SwissADME*** | | | | | |
| Gastrointestinal absorption | High |  |  |  |  |
| Blood-brain barrier (BBB) penetration | Yes |  |  |  |  |
| P-glycoprotein substrate | No |  |  |  |  |
| CYP1A2 inhibitor | Yes |  |  |  |  |
| CYP2C19 inhibitor | No |  |  |  |  |
| CYP2C9 inhibitor | No |  |  |  |  |
| CYP2D6 inhibitor | No |  |  |  |  |
| CYP3A4 inhibitor | No |  |  |  |  |
| **Experimental physico-chemical properties** | | | | | |
| Log k’w | 1.136** |  |  |  |  |
| LogS (solubility in µM) | > - 3.3** |  |  |  |  |
| Water solubility (µM) | > 500** |  |  |  |  |
| **Other** | | | | | |
| Pan Assay Interference Structure (PAINS) | 0 alerts* |  |  |  |  |

******* predicted/calculated by SwissADME [1] for SMILES: O=C(Nc1ncco1)c2cc(Cl)ncc2
**** experimentally determined by Juhás *et al.,* 2022 [2]

Log k’w – logarithm of capacity factor extrapolated to 100 % aqueous phase

**Table S3.** Prediction of human Phase I (CYP450) metabolites of AB15 by BioTransformer 3.0 [3]

| **Result_ID** | **SMILES** | **Biosystem** | **Enzyme(s)** | **Reaction** |
| --- | --- | --- | --- | --- |
| 1 | ClC1=[N+](C=CC(C(NC2=NC=CO2)=O)=C1)[O-] | Human | CYP1A2; CYP2C9; CYP2D6; CYP3A4 | **Pyridine *N*-oxidation** |
| 2 | ClC1=NC=CC(C(N(C2=NC=CO2)O)=O)=C1 | Human | CYP1A2 | ***N*-Hydroxylation** of secondary arylamide |
| 3 | ClC1=NC=C(C(C(NC2=NC=CO2)=O)=C1)O | Human | CYP1A2; CYP2E1; CYP3A4 | **Hydroxylation** of aromatic carbon para to halide group |
| 4 | ClC1=NC=CC(C(NC2=NC=CO2)=O)=C1O | Human | CYP1A2; CYP2A6; CYP2B6; CYP2C8; CYP2C9; CYP2C19; CYP2D6; CYP2E1; CYP3A4 | **Hydroxylation** of aromatic carbon ortho to halide group |
| 5 | ClC1=NC(=CC(C(NC2=NC=CO2)=O)=C1)O | Human | CYP2A6; CYP2B6; CYP2C9; CYP2C19; CYP2E1; CYP3A4 | **Hydroxylation** of aromatic carbon meta to halide group AndFromCyProduct |
| 6 | ClC1=NC=CC(C(NC2=NC=C(O2)O)=O)=C1 | Human | CYP2B6; CYP2D6; CYP2E1; CYP3A4 | **Hydroxylation** from CyProduct |
| 7 | ClC1=NC=CC(C(NC2=NC(=CO2)O)=O)=C1 | Human | CYP2B6; CYP2C9; CYP2E1 | **Hydroxylation** from CyProduct |

**Table S4.** An overview of the antibacterial activities of AB15 against selected reference strains of Gram-positive and Gram-negative bacteria. The antibacterial activity was determined by the microdilution broth method according to EUCAST recommendations. The Minimum Inhibitory Concentration (MIC) was evaluated by visual inspection, the metabolic indicator Alamar Blue, and spectrophotometric measurement. In Gram‑positive and Gram-negative bacteria, the results were read after 24 and 48-hour exposure. The data and methodology were published in Juhás *et al.,* 2022 [2].

| Bacterial strain (collection number, ATCC) | MIC (µM) AB15  24 h / 48 h | MIC (mg/L) AB15  24 h / 48 h |
| --- | --- | --- |
| *Staphylococcus aureus* subsp. a*ureus* (ATCC 29213) | 250 / 250 | 56 / 56 |
| *Staphylococcus aureus* subsp. a*ureus*, MRSA, (ATCC 43300) | 62.5 / 62.5 | 14 / 14 |
| *Staphylococcus epidermidis* (ATCC 12228) | 62.5 / 250 | 14 / 56 |
| *Enterococcus faecalis* (ATCC 29212) | 500 / 500 | 112 / 112 |
| *Escherichia coli* (ATCC 25922) | 62.5 / 250 | 14 / 56 |
| *Klebsiella pneumoniae* (ATCC 10031) | 125 / 250 | 28 / 56 |
| *Acinetobacter baumannii* (ATCC 19606) | 62.5 / 125 | 14 / 28 |
| *Pseudomonas aeruginosa* (ATCC 27853) | > 500 / > 500 | > 112 / > 112 |

*ATCC – American Type Culture Collection, MRSA – methicillin-resistant Staphylococcus aureus*

**Table S5.** An overview of the antimycobacterial activities of AB15 against selected mycobacterial strains. The antimycobacterial activity was determined by the microdilution broth method according to EUCAST recommendations. The Minimum Inhibitory Concentration (MIC) was evaluated by visual inspection, and the metabolic indicator Alamar Blue (fluorometric measurement). The results were read after exposure related to the growth of the positive control (48 to 144 h). The data and methodology were published in Juhás *et al.,* 2022 [2].

| Bacterial strain (collection code, ATCC) | MIC (µM) AB15 | MIC (mg/L) AB15 |
| --- | --- | --- |
| *Mycobacterium smegmatis* (ATCC 607) | 27.902 | 6.250 |
| *Mycobacterium aurum* (ATCC 23366) | 13.951 | 3.125 |
| *Mycobacterium avium* (ATCC 25291) | 13.951 | 3.125 |
| *Mycobacterium kansasii* (ATCC 12478) | 13.951 | 3.125 |
| *Mycobacterium tuberculosis* H37Ra (ATCC 9431) | 13.951 | 3.125 |
| *Mycobacterium tuberculosis* H37Rv (ATCC 27294) | 27.902 | 6.250 |
| *Mycobacterium tuberculosis* (IZAK) | 13.951 | 3.125 |
| *Mycobacterium tuberculosis* (MATI) | 13.951 | 3.125 |

*ATCC – American Type Culture Collection IZAK and MATI – strain designation for clinical isolates of MDR M. tuberculosis Susceptibility profile for IZAK – resistance (streptomycin, isoniazid, rifampicin, pyrazinamide), sensitivity (ethambutol) Susceptibility profile for MATI – resistance (streptomycin, isoniazid, rifampicin, pyrazinamide), sensitivity (ethambutol)*

**Table S6.** An overview of the antifungal activities of AB15 against selected reference yeast and mold strains. The antifungal activity was determined by the microdilution broth method according to EUCAST recommendations, with slight modifications. The minimum inhibitory concentration (MIC) was evaluated by visual inspection, and spectrophotometric measurement. The static incubation was performed in a humid atmosphere, at 35 ± 2 °C, for 24 and 48 h (72 and 120 h for *Trichophyton interdigitale*, respectively). The data and methodology were published in Juhás *et al.,* 2022 [2].

| **BACTERIAL STRAIN (COLLECTION NUMBER, ATCC)** | **MIC (**µM**) AB15**  **24** h **/ 48** h **(72** h **/ 120** h**)** | **MIC (MG/L) AB15**  **24** h **/ 48** h **(72** h **/ 120** h**)** |
| --- | --- | --- |
| *Candida albicans* (**ATCC 2443)** | 31.25 / 62.5 | 7 / 14 |
| *Candida krusei* (**ATCC 6258)** | 250 / 500 | 56 / 112 |
| *Candida parapsilosis* **ATCC 22019)** | 250 / 250 | 56 / 56 |
| *Candida tropicalis* (**ATCC 750)** | 500 / 500 | 112 / 112 |
| *Aspergillus fumigatus* **ATCC 204305)** | 125 / 125 | 28 / 28 |
| *Aspergillus flavus* (**CCM 8363)** | 500 / 500 | 112 / 112 |
| *Lichtheimia corymbifera* (**CCM 8077)** | 62.5 / 125 | 14 / 28 |
| *Trichophyton interdigitale* (**ATCC 953)** | 125 / 125 | 28 / 28 |

*ATCC – American Type Culture Collection, CCM – Czech Collection of Microorganism*

**Table S7.** The antibacterial activity of AB15 against Gram-positive clinical bacterial isolates. The reference internal quality control strain, *Staphylococcus aureus* ATCC 29213 and internal quality standards (ciprofloxacin, gentamicin) were also employed. The antibacterial activity was determined by the microdilution broth method according to EUCAST recommendations. The minimum inhibitory concentration (MIC) was evaluated by visual inspection, the metabolic indicator Alamar Blue, and spectrophotometric measurement. The results were read after 24 h exposure.

| Bacterial strain (ATCC/ID No.) | Clinical specimen | MIC AB15  (µM) | MIC AB15  (mg/L) |
| --- | --- | --- | --- |
| *Staphylococcus aureus* subsp*. aureus* (ATCC 29213) | Quality control | 125 - 250 | 28 - 56 |
| *Staphylococcus aureus* subsp*. aureus, methicillin-resistant* (131/16) | Throat swab | 250 | 56 |
| *Staphylococcus aureus,* vancomycin-resistant (NIPH) | ND | 125 - 250 | 28 -125 |
| *Staphylococcus capitis* (9/21) | ND | 62.5 -125 | 14 - 25 |
| *Staphylococcus haemolyticus* (11/21) | ND | 250 | 26 |

ATCC – American Type Culture Collection; CIP – ciprofloxacin; GEN – gentamicin; ID No. – internal laboratory identification number; NIPH – National Institute of Public Health, Prague

**Table S8.** Susceptibility/resistance profiles of employed bacterial clinical isolates determined by the disc diffusion and microdilution broth method according to EUCAST recommendation.

| Bacterial strain (ID No.) | Susceptibility profile |
| --- | --- |
| *Proteus mirabilis (*12/21) | R: nitrofurantoin, trimethoprim, trimethoprim-sulphonamide, cefuroxime-axetil oral, cefuroxime iv, mecillinam oral, ampicillin, cefotaxime, cefepime, ciprofloxacin, gentamicin, colistin, tigecycline; S/R: imipenem; S: amoxicillin-clavulanic acid, cefoxitin, ceftazidime, amikacin, piperacillin-tazobactam, meropenem, ceftolozane-tazobactam, ceftazidime-avibactam, temocillin |
| *Klebsiella pneumoniae* (14/21) | R: ampicillin, cefotaxime, amoxicillin-clavulanic acid, ceftazidime, cefepime, trimethoprim-sulphonamide, ciprofloxacin, piperacillin-tazobactam; S: cefoxitin, gentamicin, amikacin, colistin, imipenem, meropenem, ceftolozane-tazobactam, ceftazidime-avibactam, temocillin |
| *Klebsiella pneumoniae* (15/21) | R: ampicillin, cefotaxime, amoxicillin-clavulanic acid, ceftazidime, cefepime, trimethoprim-sulphonamide, ciprofloxacin, piperacillin-tazobactam; S: cefoxitin, gentamicin, amikacin, colistin, imipenem, meropenem, ceftolozane-tazobactam, ceftazidime-avibactam, temocillin |
| *Enterobacter cloacae* (16/21) | R: trimethoprim, trimethoprim-sulphonamide, mecillinam, amoxicillin-clavulanic acid, ampicillin, cefoxitin, cefotaxime, ceftazidime, cefepime, ciprofloxacin, gentamicin, colistin; S: amikacin, piperacillin-tazobactam, imipenem, meropenem, ceftolozane-tazobactam, ceftazidime-avibactam,  temocillin |
| *Acinetobacter baumannii* (59/16) | R: ampicillin, cefotaxime, amoxicillin-clavulanic acid, ertapenem; S: ciprofloxacin, imipenem, trimethoprim-sulphonamide, gentamicin, meropenem, amikacin |
| *Escherichia coli* (27/21) | R: ampicillin, cefotaxime, amoxicillin-clavulanic acid, cefepime, trimethoprim-sulphonamide, ciprofloxacin, piperacillin-tazobactam; S/R: ceftazidime; S: cefoxitin, gentamicin, amikacin, colistin, imipenem, meropenem, ceftolozane-tazobactam, ceftazidime-avibactam, imipenem-relebactam |
| *Acinetobacter baumannii* (20/21) | R: trimethoprim, cefuroxime iv, amoxicillin-clavulanic acid, ampicillin, cefuroxime-axetil, fosfomycin, cefotaxime, mecillinam; S/R: ciprofloxacin; S: amikacin, imipenem, meropenem, trimethoprim-sulphonamide, gentamicin |
| *Pseudomonas aeruginosa* (21/21) | R: piperacillin-tazobactam, imipenem, ciprofloxacin, ceftazidime, meropenem, levofloxacin, cefepime; S: amikacin, ceftolozane-tazobactam, ceftazidime- avibactam |
| *Enterobacter cloacae* (23/21) | R: trimethoprim, amoxicillin-clavulanic acid, ciprofloxacin, piperacillin-tazobactam, trimethoprim-sulphonamide, ampicillin, ceftazidime, gentamicin, cefotaxime, cefepime; S: ceftolozane-tazobactam, imipenem, ceftazidime-avibactam, mecillinam, amikacin, meropenem, imipenem/relebactam |
| *Escherichia coli* (24/21) | R: trimethoprim, trimethoprim-sulphonamide, cefuroxime-axetil oral, cefuroxime iv, ampicillin, cefotaxime, ceftazidime, cefepime; S: nitrofurantoin, mecillinam, fosfomycin, amoxicillin-clavulanic acid, cefoxitin, ciprofloxacin, gentamicin, amikacin, piperacillin-tazobactam, colistin, imipenem,  meropenem, ceftazidime-avibactam, imipenem |
| *Pseudomonas aeruginosa* (26/21) | R: piperacillin-tazobactam, ceftazidime, cefepime, imipenem, meropenem, amikacin, gentamicin, ciprofloxacin, levofloxacin, ceftazidime-avibactam; C: colistin, aztreonam, fosfomycin |
| *Acinetobacter baumannii* (1/23) | R: ampicillin, cefotaxime, amoxicillin-clavulanic acid, ciprofloxacin, gentamicin, amikacin, imipenem, meropenem, fosfomycin; S: trimethoprim- sulphonamide, colistin |

*ID No. – internal laboratory identification number; R – Resistant, S – Susceptible, S/R – intermediate susceptibility*

**Table S9.** The percentage of reduction of *A. baumannii* (20/21) at a 4-fold MIC concentration of AB15. The microdilution broth method and spread plate technique for colony-forming units calculation were employed for the evaluation.

| Bacterial strain (ID No.) | AB15 |  |
| --- | --- | --- |
|  | c (µM)/ 4-fold of MIC | % of reduction |
| *Acinetobacter baumannii* (20/21) | 250 | 99.983 - 99.984 % |

*ID No. – internal laboratory identification number*

**Table S10.** An evaluation of the *in vivo* toxicity of AB15 in the animal model, *Galleria mellonella*. The tested compound was administered *via* the **intra-hemocoel route**, through the last left proleg. After administration, the larvae were incubated at 37 °C for five days and inspected after 24, 48, 72, 96 and 120 h of incubation.

| Dose corresponding to mean weight of larvae per group (mg/kg of body  weight) | mg/kg of larvae body weight | | | | | | | | Control group (10 mL of PBS +  30% (v/v) DMSO | | Control group (w/o any administration) | |
| --- | --- | --- | --- | --- | --- | --- | --- | --- | --- | --- | --- | --- |
|  | **500** |  | **250** |  | **50** |  | **5** | |  |  |  |  |
| Hours (visual inspection after administration) | Number of surviving larvae | Mortality (%) | Number of surviving larvae | Mortality (%) | Number of surviving larvae | Mortality (%) | Number of surviving  larvae | Mortality (%) | Number of surviving  larvae | Mortality (%) | Number of surviving larvae | Mortality (%) |
| 0 | 8 | 0 | 8 | 0 | 8 | 0 | 8 | 0 | 8 | 0 | 8 | 0 |
| 24 | 8 | 0 | 8 | 0 | 8 | 0 | 8 | 0 | 8 | 0 | 8 | 0 |
| 48 | 7 | 12.5 | 8 | 0 | 8 | 0 | 8 | 0 | 8 | 0 | 8 | 0 |
| 72 | 7 | 12.5 | 8 | 0 | 8 | 0 | 8 | 0 | 8 | 0 | 8 | 0 |
| 96 | 7 | 12.5 | 8 | 0 | 8 | 0 | 8 | 0 | 8 | 0 | 8 | 0 |
| 120 | 7 | 12.5 | 8 | 0 | 8 | 0 | 8 | 0 | 8 | 0 | 8 | 0 |

# PBS – phosphate saline buffer, w/o administration – without administration, DMSO – dimethyl sulfoxide

**Table S11.** Evaluation of the *in vivo* toxicity of AB15 in the animal model, *Galleria mellonella,* after **per oral** administration. After administration, the larvae were incubated at 37 °C for five days and inspected after 24, 48, 72, 96 and 120 h of incubation.

| Dose corresponding to mean weight of larvae per group (mg/kg of body  weight) | mg/kg of larvae body weight | | | | | | | | Control group  (10 mL of PBS + 30% (v/v) DMSO | | Control group (w/o any administration) | |
| --- | --- | --- | --- | --- | --- | --- | --- | --- | --- | --- | --- | --- |
|  | **500** | | **250** | | **50** | | **5** | |  |  |  |  |
| Hours (visual inspection after administration) | Number of surviving larvae | Mortality (%) | Number of surviving  larvae | Mortality (%) | Number of surviving  larvae | Mortality (%) | Number of surviving  larvae | Mortality (%) | Number of surviving  larvae | Mortality (%) | Number of surviving larvae | Mortality (%) |
| 0 | 5 | 0 | 5 | 0 | 5 | 0 | 5 | 0 | 5 | 0 | 5 | 0 |
| 24 | 5 | 0 | 5 | 0 | 5 | 0 | 5 | 0 | 5 | 0 | 5 | 0 |
| 48 | 5 | 0 | 5 | 0 | 5 | 0 | 5 | 0 | 5 | 0 | 5 | 0 |
| 72 | 5 | 0 | 5 | 0 | 5 | 0 | 5 | 0 | 5 | 0 | 5 | 0 |
| 96 | 5 | 0 | 5 | 0 | 5 | 0 | 5 | 0 | 5 | 0 | 5 | 0 |
| 120 | 5 | 0 | 5 | 0 | 5 | 0 | 5 | 0 | 5 | 0 | 5 | 0 |

# PBS – phosphate saline buffer, w/o administration – without administration, DMSO – dimethyl sulfoxide

**Table S12.** Survival analyses of *Galleria mellonella* larvae after the **intra-hemocoel** administration of AB15. The values represent outputs from the pairwise comparison Log-rank Mantel-Cox test and the Hazard Ratio(s) (Mantel-Haenszel) test, with CI 95%.

| AB15 (mg/kg of body weight) | | | | |
| --- | --- | --- | --- | --- |
|  | 500 (B) | 250 (C) | 50 (D) | 5 (E) |
| 0 (control group - 30% DMSO + PBS) (A) | χ2 (1) = 1.000  p = 0.3173  A/B = 0.1353 (0.002685 to 6.821)  B/A = 7.389 (0.1466 to 372.4) | **ns**  χ2 (1) = 0.000  p > 0.9999  A/C = Undefined C/A = Undefined | **ns**  χ2 (1) = 0.000  p > 0.9999  A/D = Undefined D/A = Undefined | **ns**  χ2 (1) = 0.000  p > 0.9999  A/E = Undefined E/A = Undefined |
| 500 |  | **ns** | **ns** | **ns** |
| (B) |  | χ2 (1) = 1.000 | χ2 (1) = 1.000 | χ2 (1) = 1.000 |
|  |  | p = 0.3173 | p = 0.3173 | p = 0.3173 |
|  |  | B/C = 0.1353 (0.002685 to 6.821) | B/D = 0.1353 (0.002685 to 6.821) | B/E = 0.1353 (0.002685 to 6.821) |
|  |  | C/B =7.389 (0.1466 to 372.4) | D/B = 7.389 (0.1466 to 372.4) | E/B = 7.389 (0.1466 to 372.4) |
| 250 |  |  | **ns** | **ns** |
| (C) |  |  | χ2 (1) = 1.000 | χ2 (1) = 0.000 |
|  |  |  | p > 0.9999 | p > 0.9999 |
|  |  |  | C/D = Undefined | C/E = Undefined |
|  |  |  | D/C =Undefined | E/C =Undefined |
| 50 |  |  |  | **ns** |
| (D) |  |  |  | χ2 (1) = 0.000 |
|  |  |  |  | p > 0.9999 |
|  |  |  |  | D/E = Undefined |
|  |  |  |  | E/D = Undefined |

***ns*** *– no significant difference*

**Table S13.** Survival analyses of *Galleria mellonella* larvae after the **per oral** administration of AB15. The values represent outputs from the pairwise comparison Log-rank Mantel-Cox test and the Hazard Ratio(s) (Mantel-Haenszel) test, with CI 95%.

| AB15 (mg/kg of body weight) | | | | |
| --- | --- | --- | --- | --- |
|  | 500 (B) | 250 (C) | 50 (D) | 5 (E) |
| 0 (control group - 30% DMSO +PBS (A) | **ns**  χ2 (1) = 0.000  p > 0.9999  A/B = Undefined B/A = Undefined | **ns**  χ2 (1) = 0.000  p > 0.9999  A/C = Undefined C/A = Undefined | **ns**  χ2 (1) = 0.000  p > 0.9999  A/D = Undefined D/A= Undefined | **ns**  χ2 (1) = 0.000.  p > 0.9999  A/E = Undefined E/A = Undefined |
| 500 (B) |  | **ns**  χ2 (1) = 0.000  p > 0.9999  B/C = Undefined C/B = Undefined | **ns**  χ2 (1) = 0.000  p > 0.9999  B/D = Undefined D/B = Undefined | **ns**  χ2 (1) = 0.000  p > 0.9999  B/E = Undefined E/B = Undefined |
| 250 (C) |  |  | **ns**  χ2 (1) = 0.000  p > 0.9999  C/D = Undefined D/C = Undefined | **ns**  χ2 (1) = 0.000  p > 0.9999  C/E = Undefined E/C = Undefined |
| 50 (D) |  |  |  | **ns**  χ2 (1) = 0.000  p > 0.9999  D/E = Undefined E/D = Undefined |

***ns*** *– no significant difference*

**Table S14. – S19.** Total fractional inhibitory concentration indices (FICI) determined by checkerboard assay of ciprofloxacin (CIP) and AB15, gentamicin (GEN) and AB15, tigecycline (TGC) and AB15, trimethoprim-sulfamethoxazole (SXT) and AB15, colistin (CST) and AB15, and chloramphenicol (CHL) and AB15 combinations against *Escherichia coli* (ATCC 25922). The minimum inhibitory concentration (MIC) of the compounds alone were: MICCIP = 0.024 µM, MICAB15 = 62.5 µM in the CIP+AB15 combination. MICGEN = 2.094 µM, MICAB15 = 125 µM in the GEN+AB15 combination. MICTGC = 0.107 µM, MICAB15= 62.5 µM in the TGC+AB15 combination. MICSXT = 3.679, MIC AB15 = 62.5 µM in the SXT+AB15 combination. MICCST = 0.108 µM, MICAB15

= 125 µM in the CST and AB15 combination. MICCHL = 49.516 µM, MICAB15 = 125 µM in the CHL and AB15 combination.

| **S14. Combination of compounds (ciprofloxacin : AB15)** | | | | | |
| --- | --- | --- | --- | --- | --- |
| Concentration ratio CIP : AB15  (µM) | Concentration ratio CIP : AB15  (mg/L) | FICCIP | FICAB15 | FICI | Effect |
| 0.006 : 62.500 | 0.002 : 14.000 | 0.250 | 1.000 | 1.250 | Indifference |
| 0.012 : 62.500 | 0.004 : 14.000 | 0.500 | 1.000 | 1.500 | Indifference |
| 0.024 : 62.500 | 0.008 : 14.000 | 1.000 | 1.000 | 2.000 | Indifference |
| 0.048 : 31.250 | 0.016 : 7.000 | 2.000 | 0.500 | 2.500 | Indifference |
| 0.048 : 15.625 | 0.016 : 3.500 | 2.000 | 0.250 | 2.250 | Indifference |
| 0.048 : 7.813 | 0.016 : 1.750 | 2.000 | 0.125 | 2.125 | Indifference |
| 0.024 : 7.813 | 0.008 : 1.750 | 1.000 | 0.125 | 1.125 | Indifference |
| 0.024 : 3.906 | 0.008 : 0.875 | 1.000 | 0.063 | 1.063 | Indifference |
| 0.024 : 1.953 | 0,008 : 0.438 | 1.000 | 0.031 | 1.031 | Indifference |

| **S15. Combination of compounds (gentamicin : AB15)** | | | | | |
| --- | --- | --- | --- | --- | --- |
| Concentration ratio GEN : AB15  (µM) | Concentration ratio GEN : AB15  (mg/L) | FICGEN | FICAB15 | FICI | Effect |
| 0.033 : 62.500 | 0.016 : 14.000 | 0.016 | 0.500 | 0.516 | **Additivity** |
| 0.066 : 62.500 | 0.031 : 14.000 | 0.031 | 0.500 | 0.531 | **Additivity** |
| 0.132 : 62.500 | 0.063 : 14.000 | 0.063 | 0.500 | 0.563 | **Additivity** |
| 0.264 : 62.500 | 0.125 : 14.000 | 0.125 | 0.500 | 0.625 | **Additivity** |
| 0.528 : 62.500 | 0.250 : 14.000 | 0.250 | 0.500 | 0.750 | **Additivity** |
| 1.056 : 62.500 | 0.500 : 14.000 | 0.500 | 0.500 | 1.000 | **Additivity** |
| 2.112 : 62.500 | 1 : 14.000 | 1.000 | 0.500 | 1.500 | Indifference |
| 2.112 : 31.250 | 1 : 7.000 | 1.000 | 0.250 | 1.250 | Indifference |
| 2.112 : 15.625 | 1 : 3.500 | 1.000 | 0.125 | 1.125 | Indifference |
| 2.112 : 7.913 | 1 : 1.750 | 1.000 | 0.063 | 1.063 | Indifference |
| 2.112 : 3.906 | 1 : 0.875 | 1.000 | 0.031 | 1.031 | Indifference |
| 2.112 : 1.9531 | 1 : 0.438 | 1.000 | 0.016 | 1.016 | Indifference |

| **S16. Combination of compounds (tigecycline : AB15)** | | | | | |
| --- | --- | --- | --- | --- | --- |
| Concentration ratio TGC : AB15  (µM) | Concentration ratio TGC : AB15  (mg/L) | FICTGC | FICAB15 | FICI | Effect |
| 0.002 : 62.500 | 0.001 : 14.000 | 0.016 | 1.000 | 1.016 | Indifference |
| 0.003 : 62.500 | 0.002 : 14.000 | 0.031 | 1.000 | 1.031 | Indifference |
| 0.007 : 62.500 | 0.004 : 14.000 | 0.063 | 1.000 | 1.063 | Indifference |
| 0.013 : 62.500 | 0.008 : 14.000 | 0.125 | 1.000 | 1.125 | Indifference |
| 0.027 : 62.500 | 0.016 : 14.000 | 0.250 | 1.000 | 1.250 | Indifference |
| 0.053 : 62.500 | 0.031 : 14.000 | 0.500 | 1.000 | 1.500 | Indifference |
| 0.107 : 62.500 | 0.063 : 14.000 | 1.000 | 1.000 | 2.000 | Indifference |
| 0.107 : 31.250 | 0.063 : 7.000 | 1.000 | 0.500 | 1.500 | Indifference |
| 0.107 : 15.625 | 0.063 : 3.500 | 1.000 | 0.250 | 1.250 | Indifference |
| 0.107 : 7.813 | 0.063 : 1.750 | 1.000 | 0.125 | 1.125 | Indifference |
| 0.107 : 3.906 | 0.063 : 0.875 | 1.000 | 0.063 | 1.063 | Indifference |
| 0.107 : 1.953 | 0.063 : 0.438 | 1.000 | 0.031 | 1.031 | Indifference |

| **S17. Combination of compounds (trimethoprim-sulfamethoxazole : AB15)** | | | | | |
| --- | --- | --- | --- | --- | --- |
| Concentration ratio SXT : AB15  (µM) | Concentration ratio SXT : AB15  (mg/L) | FICSXT | FICAB15 | FICI | Effect |
| 0.057 : 62.500 | 0.031 : 14.000 | 0.016 | 1.000 | 1.016 | Indifference |
| 0.115 : 62.500 | 0.062 : 14.000 | 0.031 | 1.000 | 1.031 | Indifference |
| 0.230 : 62.500 | 0.125 : 14.000 | 0.063 | 1.000 | 1.063 | Indifference |
| 0.460 : 62.500 | 0.250 : 14.000 | 0.125 | 1.000 | 1.125 | Indifference |
| 0.920 : 62.500 | 0.500 : 14.000 | 0.250 | 1.000 | 1.250 | Indifference |
| 1.839 : 62.500 | 1 : 14.000 | 0.500 | 1.000 | 1.500 | Indifference |
| 3.679 : 62.500 | 2 : 14.000 | 1.000 | 1.000 | 2.000 | Indifference |
| 7.358 : 31.250 | 4 : 7.000 | 2.000 | 0.500 | 2.500 | Indifference |
| 7.358: 15.625 | 4 : 3.500 | 2.000 | 0.250 | 2.250 | Indifference |
| 7.358: 7.813 | 4 : 1.750 | 2.000 | 0.125 | 2.125 | Indifference |
| 7.358 : 3.906 | 4 : 0.875 | 2.000 | 0.063 | 2.063 | Indifference |
| 7.358 : 1.953 | 4 : 0.438 | 2.000 | 0.031 | 2.031 | Indifference |

| **S18. Combination of compounds (colistin : AB15)** | | | | | |
| --- | --- | --- | --- | --- | --- |
| Concentration ratio CST : AB15  (µM) | Concentration ratio CST : AB15  (mg/L) | FICCST | FICAB15 | FICI | Effect |
| 0.027 : 62.500 | 0.031 : 14.000 | 0.250 | 0.500 | 0.750 | **Additivity** |
| 0.054 : 62.500 | 0.063 : 14.000 | 0.500 | 0.500 | 1.000 | **Additivity** |
| 0.108 : 62.500 | 0.125 : 14.000 | 1.000 | 0.500 | 1.500 | Indifference |
| 0.216 : 31.250 | 0.250 : 7.000 | 2.000 | 0.250 | 2.250 | Indifference |
| 0.216 : 15.625 | 0.250 : 3.500 | 2.000 | 0.125 | 2.125 | Indifference |
| 0.216 : 7.813 | 0.250 : 1.750 | 2.000 | 0.063 | 2.063 | Indifference |
| 0.216 : 3.906 | 0.250 : 0.875 | 2.000 | 0.031 | 2.031 | Indifference |
| 0.216 : 1.953 | 0.250 : 0.438 | 2.000 | 0.016 | 2.016 | Indifference |

| **S19. Combination of compounds (chloramphenicol : AB15)** | | | | | |
| --- | --- | --- | --- | --- | --- |
| Concentration ratio CHL : AB15  (µM) | Concentration ratio CHL : AB15  (mg/L) | FICCHL | FICAB15 | FICI | Effect |
| 0.097 : 125.000 | 0.031 : 28.000 | 0.002 | 1.000 | 1.002 | Indifference |
| 0.193 : 125.000 | 0.063 : 28.000 | 0.004 | 1.000 | 1.004 | Indifference |
| 0.387 : 125.000 | 0.125 : 28.000 | 0.008 | 1.000 | 1.008 | Indifference |
| 0.774 : 125.000 | 0.250 : 28.000 | 0.016 | 1.000 | 1.016 | Indifference |
| 1.547 : 125.000 | 0.500 : 28.000 | 0.031 | 1.000 | 1.031 | Indifference |
| 3.095 : 62.500 | 1.000 : 14.000 | 0.063 | 0.500 | 0.563 | **Additivity** |
| 6.189 : 125.000 | 2.000 : 28.000 | 0.125 | 1.000 | 1.125 | Indifference |
| 12.379 : 62.500 | 4.000 : 14.000 | 0.250 | 0.500 | 0.750 | **Additivity** |
| 24.758 : 62.500 | 8.000 : 14.000 | 0.500 | 0.500 | 1.000 | **Additivity** |
| 49.516 : 31.250 | 16.000 : 7.000 | 1.000 | 0.250 | 1.250 | Indifference |
| 49.516 : 15.625 | 16.000 : 3.500 | 1.000 | 0.125 | 1.125 | Indifference |
| 49.516 : 7.813 | 16.000 : 1.750 | 1.000 | 0.063 | 1.063 | Indifference |
| 49.516 : 3.906 | 16.000 : 0.875 | 1.000 | 0.031 | 1.031 | Indifference |
| 49.516 : 1.953 | 16.000 : 0.438 | 1.000 | 0.016 | 1.016 | Indifference |

*FIC – fractional inhibitory concentration, FICI – fractional inhibitory concentration index, FIC*CIP, GEN, TGC, SXT, CST, CHL, AB15 *= MIC of the combination / MIC*CIP, GEN, TGC, SXT, CST, CHL *alone, FICI = FIC*CIP,

GEN, TGC, SXT, CST, CHL *+ FIC*AB15*. The effect was interpreted as follows: synergy when FICI ≤ 0.5, an additive effect when 0.5 < FICI ≤1, an indifferent effect when 1 < FICI ≤ 4, an antagonistic effect when FICI > 4.*


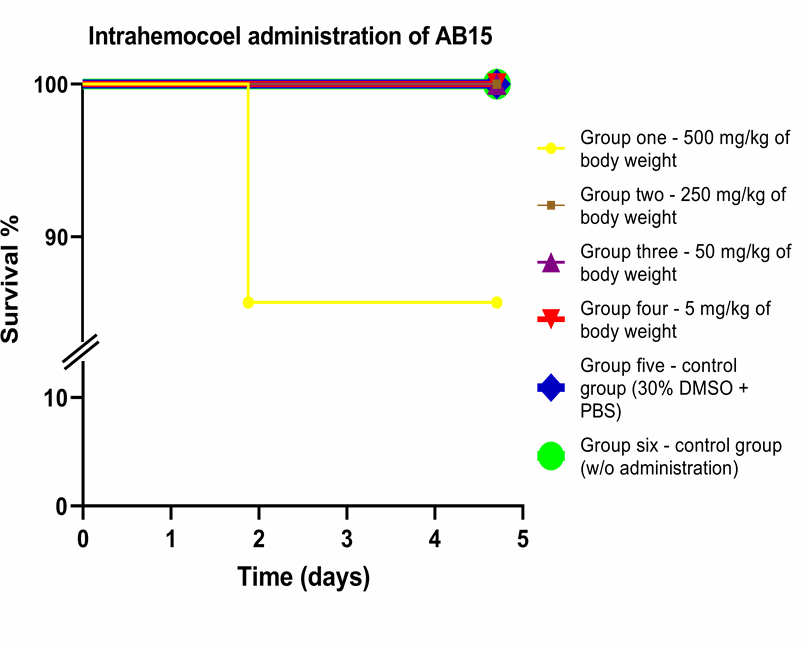


**Figure S3.** Survival curves of animal model, *Galleria mellonella*, after **intra-hemocoel** administration of AB15. After administration, larvae were incubated at 37°C for five days and inspected after 24, 48, 72, 96 and 120 h of incubation.


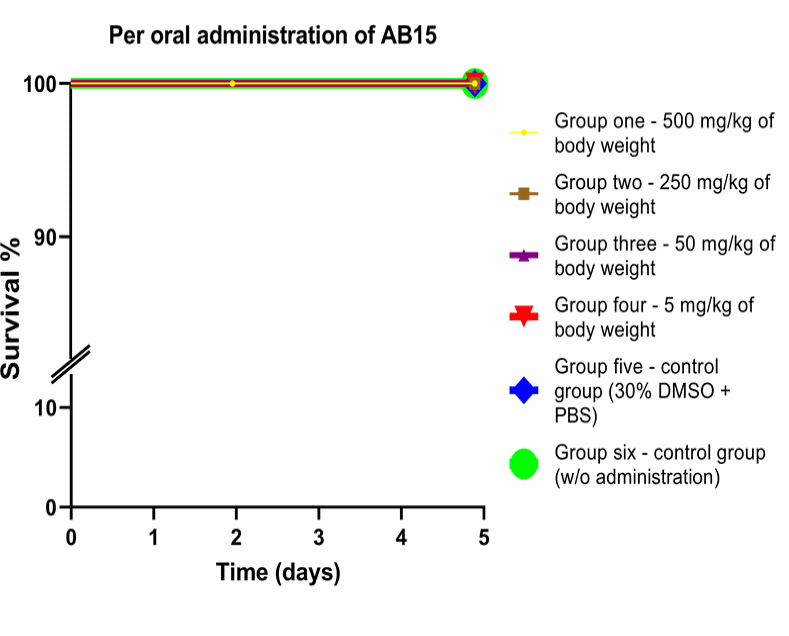


**Figure S4.** Survival curves of animal model, *Galleria mellonella*, after per oral administration of AB15. After administration, larvae were incubated at 37°C for five days and inspected after 24, 48, and 120 h of incubation.

## REFERENCES

1. Daina A, Michielin O, Zoete V. SwissADME: a free web tool to evaluate pharmacokinetics, drug-likeness and medicinal chemistry friendliness of small molecules. Sci Rep-Uk. 2017;7. **DOI:** ARTN 4271710.1038/srep42717.
2. Juhas M, Bachtikova A, Nawrot DE, Hatokova P, Pallabothula VSK, Diepoltova A, Jandourek O, Barta P, Konecna K, Paterova P, Sestak V, Zitko J. Improving Antimicrobial Activity and Physico- Chemical Properties by Isosteric Replacement of 2-Aminothiazole with 2-Aminooxazole. Pharmaceuticals (Basel). 2022;15(5). **DOI:** 10.3390/ph15050580.
3. Wishart DS, Tian S, Allen D, Oler E, Peters H, Lui VW, Gautam V, Djoumbou-Feunang Y, Greiner R, Metz TO. BioTransformer 3.0-a web server for accurately predicting metabolic transformation products. Nucleic Acids Res. 2022;50(W1):W115-W23. **DOI:** 10.1093/nar/gkac313.
