# Supplementary material for: A new bactericidal chlorinated derivative containing 2-aminooxazole potentiates antibacterial action of colistin against multidrug-resistant acinetobacter baumannii
Source: Med Microbiol Immunol. 2025 Sep 19;214(1):44. doi: 10.1007/s00430-025-00854-y (PMC12449380; doi:10.1007/s00430-025-00854-y)
Supplement: Supplementary file 2 — Supplementary file1 (DOCX 14 kb) [file 430_2025_854_MOESM2_ESM.docx]

**SUPPLEMENTARY INFORMATION**

Data outputs from nuclear magnetic resonance spectroscopy (NMR), infrared spectroscopy (IR), high-resolution mass spectroscopy (HRMS), and high-performance liquid chromatography (HPLC); Antibacterial activity of internal quality standards (ciprofloxacin and gentamicin) against clinical bacterial isolates (Table S1); Selected physico-chemical and pharmacokinetic parameters of candidate compound AB15 (Table S2); Prediction of human Phase I (CYP450) metabolites of AB15 (Table S3); An overview of antibacterial activities of AB15 against reference Gram-positive and Gram-negative bacteria (Table S4); An overview of antimycobacterial activities of AB15 against mycobacterial strains (Table S5); An overview of antifungal activities of AB15 against selected reference yeast and mold strains (Table S6); Antibacterial activity of AB15 against Gram-positive clinical bacterial isolates (Table S7); Susceptibility/resistance profiles of employed bacterial clinical isolates (Table S8); Percentual reduction of clinical isolate *Acinetobacter baumannii* designated (20/21) in 4-fold MIC concentration of AB15 (Table S9); Evaluation of *in vivo* toxicity of AB15 in animal model, *Galleria mellonella,* after intra-hemocoel administration (Table S10); Evaluation of *in vivo* toxicity of AB15 in animal model, *Galleria mellonella,* after per oral administration (Table S11); Survival analyses of *Galleria mellonella* larvae after intra-hemocoel administration of AB15 (Table S12); Survival analyses of *Galleria mellonella* larvae after per oral administration of AB15 (Table S13); Total fractional inhibitory concentration indices (FICI) determined by checkerboard assay of AB15 and CIP, GEN, TGC, SXT, CST, and CHL combination against *E. coli* (ATCC 25922) (Tables S14-S19); ^1^H NMR (top) and ^13^C NMR (bottom) spectra of compound AB15 (Figure S1); HPLC chromatogram of compound AB15 (purity 99.4% at 254 nm) (Figure S2); Survival curves of animal model, *Galleria mellonella*, after intra-hemocoel administration of AB15 (Figure S3); Survival curves of animal model, *Galleria mellonella*, after per oral administration of AB15 (Figure S4).
